# Governing Tripolye: Integrative architecture in Tripolye settlements

**DOI:** 10.1371/journal.pone.0222243

**Published:** 2019-09-25

**Authors:** Robert Hofmann, Johannes Müller, Liudmyla Shatilo, Mykhailo Videiko, René Ohlrau, Vitalii Rud, Nataliia Burdo, Marta Dal Corso, Stefan Dreibrodt, Wiebke Kirleis

**Affiliations:** 1 Institute for Pre- and Protohistory, Kiel University, Kiel, Schleswig-Holstein, Germany; 2 Collaborative Research Centre 1266: "Scales of Transformation—Human-Environmental Interaction in Prehistoric and Archaic Societies", Kiel University, Kiel, Schleswig-Holstein, Germany; 3 Laboratory of Archaeology, Borys Grinchenko Kyiv University, Kyiv, Ukraine; 4 Institute of Archaeology, National Academy of Sciences of Ukraine, Kyiv, Ukraine; 5 Institute for Ecosystem Research, Kiel University, Kiel, Schleswig-Holstein, Germany; University at Buffalo - The State University of New York, UNITED STATES

## Abstract

Recently, high-resolution magnetometry surveys have led to the discovery of a special category of buildings–so-called ‘mega-structures’–situated in highly visible positions in the public space of Tripolye giant-settlements of the late 5^th^ and first half of the 4^th^ millennium BCE. In this paper we explore what these buildings actually are and how they can contribute to the understanding of the development of social space in Tripolye giant-settlements. For this investigation, we linked newly obtained excavation data from the giant-settlement Maidanetske, Ukraine, with a much larger sample of such buildings from magnetic plans obtained in the region between the Carpathian foothills and the Dnieper River. Accordingly, Tripolye mega-structures represent a particular kind of integrative building documented in many non-ranked ethnographic contexts. Based on our results we are interpreting that these buildings were used for various ritual and non-ritual activities, joint decision-making, and the storage and consumption of surplus. In Tripolye giant-settlements at least three different categories of mega-structures could be identified which most likely represent different levels of socio-political integration and decision-making. The emergence of this hierarchical system of high-level integrative buildings for the whole community and different low-level integrative architectures for certain segments of local communities was related to the rise of Tripolye mega-sites. The presence of different integrative levels most likely reflects the fusion of different previously independent communities in the giant-settlements. Later in the mega-site development, we observe how low-level integrative buildings increasingly lose their importance indicated by shrinking size and, finally, their disappearance. This observation might indicate that the power which was previously distributed across the community was transferred to a central institution. It is argued that the non-acceptance of this concentration of power and the decline of lower decision-making levels might be a crucial factor for the disintegration of Tripolye giant-settlements around 3600 BCE.

## Introduction

**Fig 1:**
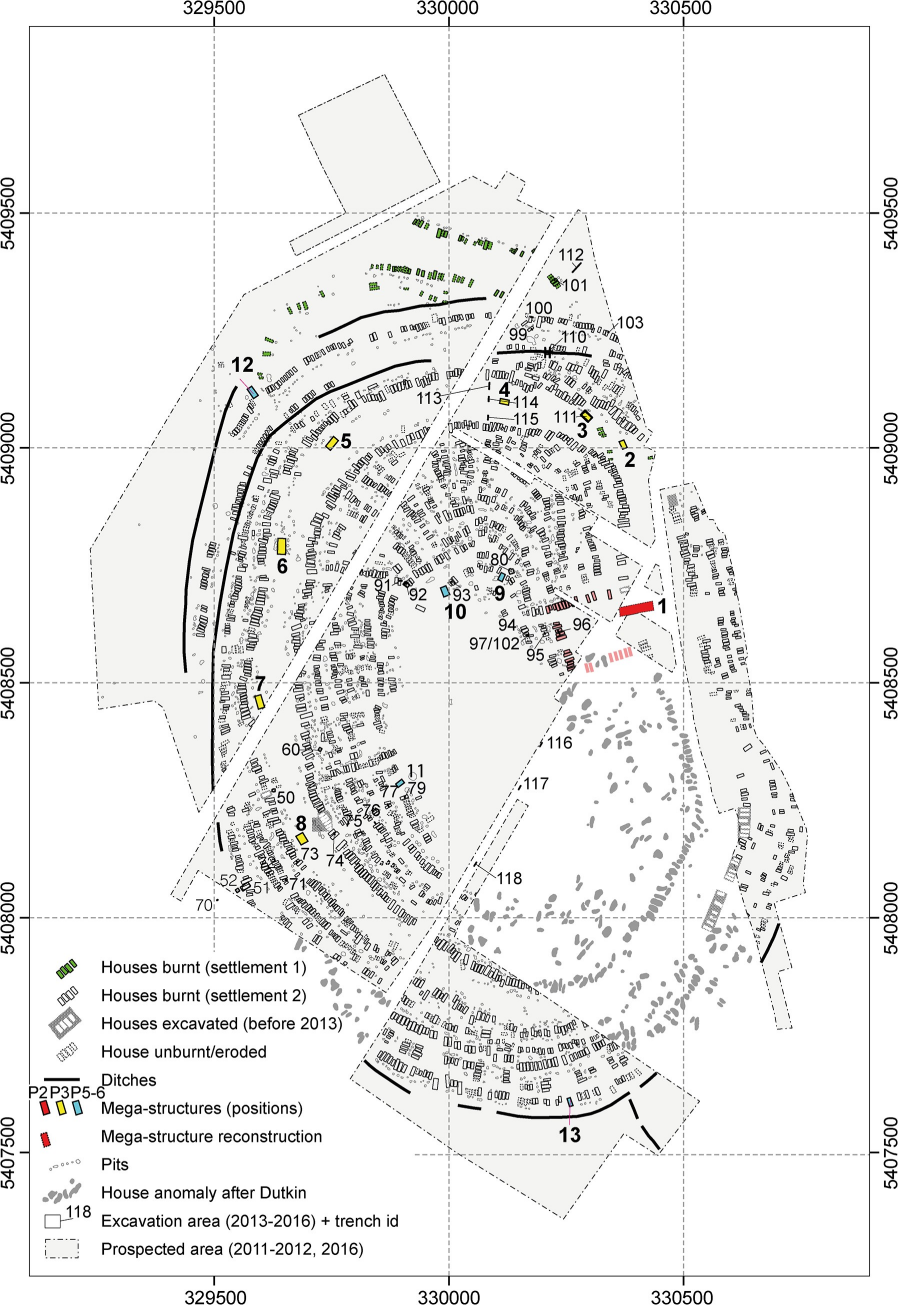
Maidanetske. **Redrawing of the plan of the magnetic survey (Adapted and extended for publication after Fig 22a of [7] under CC BY licence with permission from the Institute of Pre- and Protohistoric Archaeology, Kiel University, Germany).** Green buildings: Dwellings of settlement Maidanetske 1. White buildings: Dwellings of settlement Maidanetske 2. Light red buildings: adjacent dwellings of the primary plaza. Red buildings: Mega-structures at the primary plaza. Yellow buildings: Mega-structures in the ring-corridor. Blue buildings: Mega-structures at different positions of radial pathways.

Between ca. 4100–3600 BCE ‘giant-settlements’ or ‘mega-sites’ with thousands of houses arranged in a very specific centripetal layout emerged in a concentrated area of the Southern Bug-Dnieper Interfluve in the eastern part of the Tripolye distribution area (Fig 1). Currently, these unique settlements are the focus of different international projects [1–6]. Interpretations of their nature span between two extremes, from early urban phenomenon with complex social stratification to seasonally used pilgrimage sites. Thus, the question of their social organisation is newly under discussion [7–14].

Empirical anthropological research suggests that additional political institutions become necessary with rising population sizes in order to manage increased social complexity and scalar stress in large population agglomerations [15, 16]. Thus, the question arises how might these large Chalcolithic settlements have been organised in socio-political terms? While evolutionary-thinking authors assume a strong correlation between population sizes, organisational complexity, and social stratification [17], other authors stress that group size alone represents an insufficient criterion to predict social organisation. Rather, different modes of human cooperation have to be considered [18, 19]. Both archaeologists [20] and ethnographers [21, 22] have described the many possibilities of social organization from non-stratified to stratified societies existing independently from demographic, economic, and technological preconditions.

In Tripolye societies, no clear indicators of pronounced wealth inequality, social stratification, or hierarchies have been identified so far, neither at the local level of large sites nor at the regional level between neighbouring villages [14, 23–25]. Consistent with that is the high degree of standardization of the houses and their furnishing. Thus, we assume [16] a rather balanced, non-hierarchical, corporative and negotiation-based mode of social organisation [12].

According to ethnographic sources, such non-stratified societies are frequently associated with integrative architecture or other integrative facilities (e.g. public squares); within these spaces, integrative activities and joint decision-making take place as mechanisms to maintain the social balance [26–28]. In many societies different levels of integration–for some part of the local community, for the whole local community, or even at a regional level–can be identified. The architecture mirrors the differentiated social organisation of these societies and low- and high-level social integration mechanisms.

The suggestion that in Tripolye mega-sites a hierarchy of public buildings existed was supposed first in 1987 by Mycola Shmaglij and Mykhailo Videiko [29] based on the comparison of different buildings in Maidanetske. A completely new category of large buildings characterised by their highly visible positioning in public spaces was discovered through high-resolution magnetic surveys over the last decade [7–10, 30, 31]. Since these so called ‘mega-structures’ might represent examples of such ‘integrative architecture’, their importance for the understanding of Tripolye societies can hardly be overestimated.

There is a broad consensus that mega-structures represent some kind of public buildings [2, 8, 10, 31] and that they reflect the social organisation and the fused character of Tripolye giant-settlements [8, 10, 11, 32]. However, there is disagreement regarding the actual activities which were performed in such buildings [30]. Partly, this is due to the still very limited evidence related to the function of these buildings from excavations. So far questions concerning the temporal development of such buildings in relation to the formation of mega-sites and their spatial variations within in the large Tripolye distribution area remain unanswered.

The main aim of this paper is to clarify what the mega-structures actually are and how these buildings can contribute to the reconstruction of the social organization in Tripolye settlements and the understanding of the mega-site phenomenon. Before we will discuss these questions in the last section of this article, different aspects need to be explored:

1. In order to understand the nature of mega-structures, it is necessary to reconstruct their setup and define what differentiates them from domestic dwellings.
2. In order to identify different decision-making levels, categories of mega-structures recognizable in the archaeological record must be established as well as the extent to which they represent similar or different phenomena.
3. In order to identify different scales of integration, the number of people potentially using mega-structures in relation to the total population of Tripolye settlements must be determined.
4. In order to identify social processes and their spatial conditioning it is necessary to investigate how the mega-structures developed over time and vary within sites, between sites and across larger geographic spaces.

When these different aspects are synthesized, mega-structures represent a key-element for social-historical reconstructions of Tripolye societies.

## Data and methods

### Analytical steps

In order to answer the outlined research questions, data from different excavations and magnetic surveys were included into our analyses. The data was analysed on multiple scales:

1. At the local level of the giant-settlement Maidanetske, mega-structure 3 (Fig 1) was excavated and analysed in detail as an example of the architectural design, the construction method, and activity areas within such a building.
2. The results from this detailed investigation were linked with information about twelve other mega-structures across the site in order to test the scope and validity of the results obtained.
3. Also at the level of the site, in order to evaluate to what extent mega-structures are different from domestic dwellings, the architecture and find inventory of mega-structure 3 was compared qualitatively and quantitatively with that of normal dwellings.
4. On a meso-regional and macro-regional level, 104 mega-structures from high resolution magnetic maps of 19 settlements and excavations were analysed with regard to their frequency, location, dimensions, and architectural characteristics in order to detect their architectural variability and to identify different categories of mega-structures.
5. In conjunction with other information extracted from the analysed magnetic plans, estimations of the ‘use group size’ of mega-structures were performed to identify the groups of people in terms of population size who potentially used the buildings. The term ‘use group’ was introduced by Michael Adler [26] for an cross-cultural ethnographic sample and refers to the population of the local group most intimately associated with the use of an integrative building.
6. This regional data set was further analysed so as to draw basic development lines of mega-structures and to detect their regional variability.
7. The synthesis of the different aspects finally allows a general statement on mega-structures in the discussion section of this article.

### Terminology and criteria for the identification of mega-structures

The first Tripolye mega-structures were identified in 2009 during high-resolution magnetic surveys [33]. The application of improved survey techniques led to the discovery of rectangular constructions bigger than normal houses located mainly in non-built spaces of the domestic sites that were not detectable in former magnetic surveys because of their lower resolution.

The term ‘mega-structure’ was introduced by Mykhailo Videiko and John Chapman for a large construction that was investigated in Nebelivka in 2012 [33]. Within our research at Maidanetske, the term was adopted and used for all large buildings in highly visible positions. They could be identified mainly in otherwise unbuilt concentric ring-corridors of the giant-settlement, which we interpret as public areas in-between residential domestic zones [7, 8]. In addition to this first, most important criterion, two other criteria are hierarchically used in the identification of mega-structures in magnetic site plans. Namely, these buildings display specific architecture in comparison to domestic dwellings and they often have extraordinary dimensions. Due to the hierarchy of our three criteria, in some cases very small buildings are classified as ‘mega-structures’ if they are located in certain prominent positions. The number of mega-structures is many times lower than that of residential houses.

According to René Ohlrau, a distinction need to be made between *‘giant mega-structures*’ which occur only once per settlement and the more frequent ‘*ring- or pathway mega-structures*’ [34]. Giant mega-structures are buildings up to 70 m in length and 24 m in width located in very specific rectangular unbuilt ‘*plazas*’ situated at the northern, north-eastern or eastern side of settlements close to their main entrance. A further terminological distinction is necessary between a ‘primary plaza’ located at the main entrance to the site and occurring once per settlement, and ‘secondary plazas’, which can be found repeatedly at different locations in the settlement. In contrast, multiple ring- or pathway-mega-structures were found in the public spaces of settlements of the Southern Bug-Dnieper interfluve. For this paper, we continue to use the relatively general phrase ‘mega-structure’ as an umbrella term so as to include both types of mega-structures in our analyses.

### Materials

#### Local data base: Maidanetske

With a size of ca. 200 ha, Maidanetske is one of the largest Tripolye sites in the Southern Bug-Dnieper interfluve (Fig 1). Excavations at the site took place in the 1970s and 1980s and again from 2011–2016 [35–37]. According to a large series of ^14^C dates, the settlement was occupied over period of 350 years ca. 3990–3640 BCE [25, 34]. Based on the dominating pottery style, Maidanetske belongs to the Tomashovka local group of Tripolye C1. The site is characterised by concentric rings of about 3000 houses with associated pits around a large unbuilt space. An unbuilt concentric ring-corridor ca. 100 m wide in-between the house-ring areas and pathways, which are leading radially into the site crossing the house-rings, are also present. Beside houses and associated pits, other features have been detected, namely an enclosure made up of segmented elongated pits and pottery kilns. Like the plazas, the central space empty of houses, the radial paths, and the concentric ring-corridor are interpreted as public areas and communication paths.

In Maidanetske, thirteen magnetic features distributed across the entire settlement were identified as mega-structures according to the above mentioned three criteria (Fig 1). Mega-structure 3 was chosen for excavation in 2016 to investigate a representative mega-structure within the ring-corridor of the settlement. For comparison of mega-structures with domestic houses, the dwellings 44 and 59 from Maidanetske were used, which were excavated in the same manner [34, 37].

#### Regional data: Mega-structures from magnetic surveys and excavations

**Fig 2:**
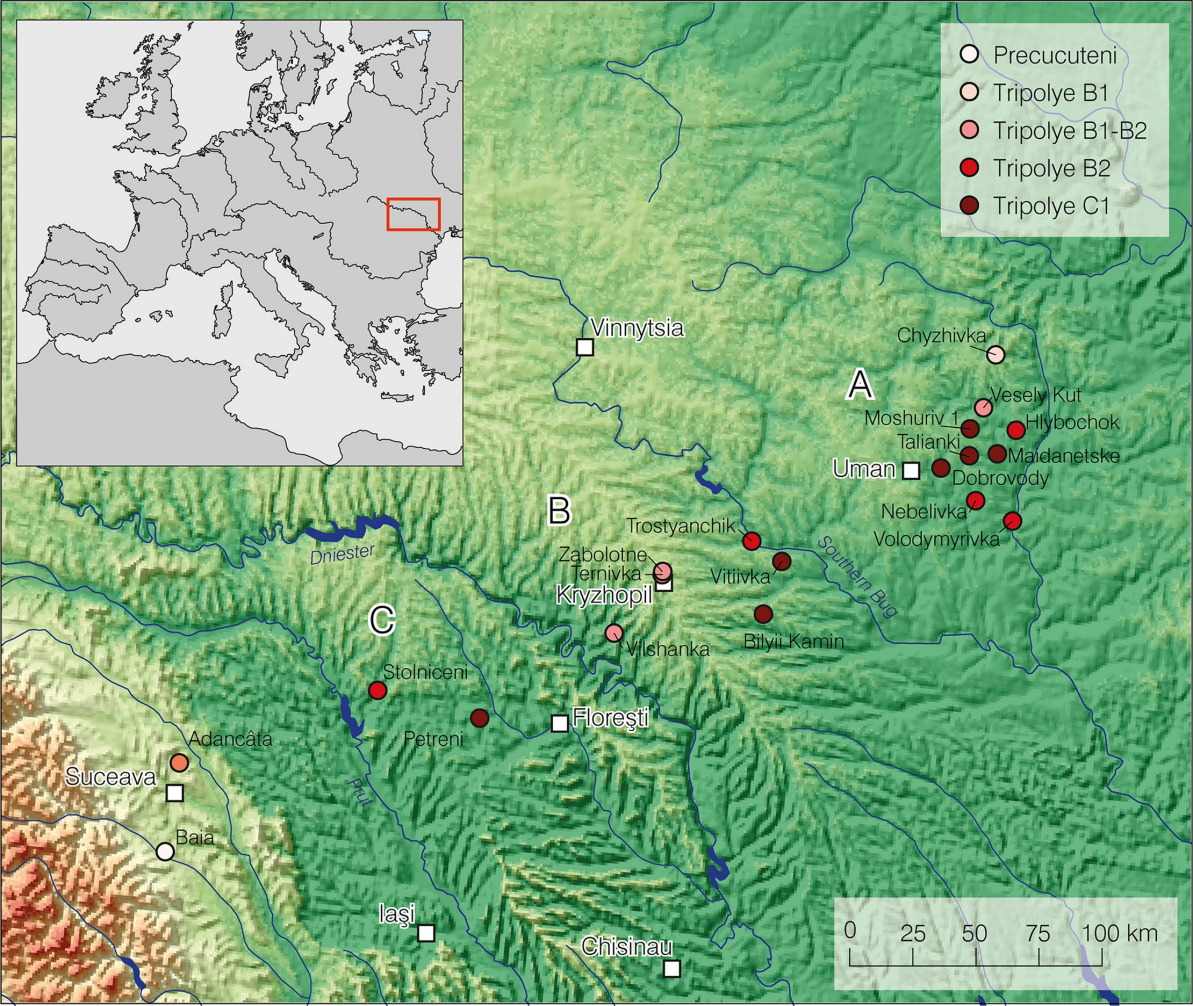
Map of sites with mega-structures analysed in this article and Tripolye periods.

High resolution magnetic plans provide detailed information on a large sample of 104 mega-structures from 19 settlements (Tables 1 and 2). Geographically this sample covers three regions: the Southern Bug-Dnieper interfluve, the Southern Bug-Dniester interfluve, and the Middle Dniester region (Fig 2). Chronologically, the sample includes sites from the periods Tripolye A to Tripolye C1–C2, i.e. from ca. 4600–3600 BCE. The regional dataset allows an investigation of general characteristics of mega-structures. The sample include three other excavated mega-structures in Baia [38], Nebelivka [30, 31], and Dobrovody [39] which provide more detailed information regarding architecture and furnishing and helps to evaluate the validity of the data obtained in Maidanetske.

**Table 1:** List of sites with mega-structures analysed in this article with information regarding location (latitude/longitude), dating, size, number of houses, average house size, number of mega-structures, estimations of use group sizes, and references. Column ‘phase’: PC = Precucuteni, T = Tripolye, C = Cucuteni. Column ‘reference’: u = unpublished. * rounded.

site	latitude	longitude	study region	phase	size (ha)	count houses	settlement area measured (ha)	percentage measured/preserved	count/estimation houses total	count high level mega-structures	count low-level mega-structures	total number of mega-structures	settlement area/mega-structure (ha)	count houses/high-level mega-structure	count houses/low-level mega-structure	average house size	use group size high level	average use group size low level*	reference
Adâncata	47.758220	26.285533	C	C-A-B1	1.5	?	1.5	100	?	1	0	1	2	?	0	35.5	150–185		[40]
Baia	47.440981	26.215325	C	PC	1.95	?	1.95	100	25.4	1	0	1	2	25.4	0	28.6	160–200		[38, 40]
Bilyi Kamin	48.266356	29.397327	B	T-C1	97	429	40.6	42	1020	3	1	4	10	340		55.8	2710–8130		[41]
Chyzhivka	49.156769	30.703630	A	T-B1	20	80	11.6	58	138	1	0	1	12	138	0	70.4	1390		u
Dobrovody	48.762810	30.378585	A	T-C1	211	296	24	14	2200	?	4–5	5	5	?	59–74	70.3		590–740	[7]
Hlybochok	48.884746	30.793430	A	T-B2	130	85	10.9	8.4	1500	3	1	4	3	500	78				[8]
Maidanetske	48.804105	30.685547	A	T-C1	200	2000	138	69	3000	1	12	13	11	?	167	72.3	20660	1725	[7]
Moshuriv 1	48.897648	30.545502	A	T-C1	7	84	7	100	84	1	3	4	2	84	28	70.0	840	280	[8]
Nebelivka	48.640990	30.557135	A	T-B2	235	1418	235	100	1450	1	19	20	12	1450	76	70.0	14500	760	[42]
Petreni	47.916771	27.876372	C	T-B2-C1	25	454	22.5	90	454	1	12	13	2	454	38	65.0	4215	350	[43]
Stolniceni	48.018772	27.336072	C	T-B2	33	350	33	100	350	2	0	2	17	175	0		1750–3500		[5]
Talianky	48.801938	30.534719	A	T-C1 late	320	1335	224	70	2220	?	9	9	25	?	148	69.8		1480	[7]
Ternivka	48.416875	28.862549	B	T-B1-B2	8	56	6	75	76	1	0	1	6	56	0	76.0	825		u
Trostianchyk	48.527579	29.3474463	B	T-B2	3	48	2.55	85	57	1–2	0	1	3	0	28–56	53.9	215–430		[44]
Veselyi Kut	48.970509	30.623021	A	T-B1-B2	60	257	33.1	55	467	1	[1/5]	6	6	467	51	62.6	4180		u
Vilshanka	48.212504	28.598318	B	T-B1-B2	53	101	42.4	80	126	1	0	1	42	125		57.7	1040		u
Viitivka	48.452057	29.504841	B	T-C1	48	38	9.7		400	3	0	3	3	133		78.0	1490–4460		[8]
Volodymyrivka	48.562180	30.749858	A	T-B2	60	331	23.7	40	830	1	10	11	2	[830]	33	66.6	2630–7890	310	u
Zabolotne	48.421087	28.865429	B	T-B1-B2	37	382	29.1	78	490	2	0	2	15	245	0	87.0	6090		u

**Table 2:** List of mega-structures analysed in this article containing information regarding floor area, dimensions, type affiliation, interior division, furnishing, and position in the settlement.

site	Id	region	phase	criterion size	criterion position	criterion architecture	criterion count	floor area (m^2^)	length	width	length to width ratio	type	interpretation	type architecture, unbuilt interior space	division in longitudinal dir.	central installation	Position (“main” mega-structure)	position
Adâncata	Ada-1	C	C-A-B1	1	1	1	3	240.0	20.0	12.0	1.7	6	high	empty	two-part?	?		2
Baia	Bai-1	C	PC	1	1	1	3	195.4	17.6	11.1	1.6	4?	high	full	multi-part	?		1
Bilyi Kamin	BK-1	B	T-C1	1	1		2	381.0	36.0	10.0	3.6	8a	high	full	two-part	?	NE	2
Bilyi Kamin	BK-2	B	T-C1	1	1		2	>376.0	>47.0	8.0		8a	high	full	two-part	?	NE	2
Bilyi Kamin	BK-3	B	T-C1	1	1		2	733.0	64.0	11.5	5.6	8b	high	full	two-part	?	NE	2
Bilyi Kamin	BK-4	B	T-C1	1	1	1	3	381.0	29.0	11.0	2.6	6a	low	empty	two-part	no		3
Chyzhivka	Chy-1	A	T B1		1	1	2	175.0	19.0	10.0	1.9	3	high	empty	one part	?	ENE	2
Dobrovody	Dob-1	A	T-C1			1	1			14.9		2	low	empty	one part?	yes		5
Dobrovody	Dob-2	A	T-C1			1	2	278.0	25.5	10.9	2.3	2c	low	empty	one part	yes		4
Dobrovody	Dob-3	A	T-C1	1		1	2	586.3	33.5	17.5	1.9	2c	low	empty	one part	yes		5
Dobrovody	Dob-4	A	T-C1	1	1	1	3	1209.0	46.5	26.0	1.8	2b	low	empty	one part	yes		3
Dobrovody	Dob-5	A	T-C1		1		1	212.0	21.2	10.0	2.1	2	dwelling	empty	one part	?		4
Hlybochok	Hly-1	A	T-B2	1	1	1	3	315.0	30.0	10.5	2.9	6b	high	empty	two-part	no	N	2
Hlybochok	Hly-2	A	T-B2	1	1	1	3	670.0	51.5	13.0	4.0	9	high	full	two-part	?	N	2
Hlybochok	Hly-3	A	T-B2	1	1	1	3	178.5	21.0	8.5	2.5	6b	high	empty	two-part	?	NNE	2
Hlybochok	Hly-4	A	T-B2	1	1	1	3	356.5	23.0	15.5	1.5	2b	low	empty	one part	yes		3
Maidanetske	Mai-1	A	T-C1		1	1	2	>312.0	>26.0	>12.0		?	high	empty	?	?		2
Maidanetske	Mai-2	A	T-C1		1	1	2	175.0	17.5	10.0	1.8	2b	low	empty	one part	yes		3
Maidanetske	Mai-3	A	T-C1		1	1	2	155.0	18.0	8.6	2.1	3	low	partly empty	two-part?	yes		3
Maidanetske	Mai-4	A	T-C1		1	1	2	180.0	18.0	10.0	1.8	2b	low	empty	one part?	yes?		3
Maidanetske	Mai-5	A	T-C1		1		1	378.0	27.0	14.0	1.9	5a	low	full	?	?		3
Maidanetske	Mai-6	A	T-C1		1	1	2	578.0	34.0	17.0	2.0	2a	low	empty	one/two-part?	?		3
Maidanetske	Mai-7	A	T-C1		1		1	391.0	29.0	13.5	2.1	6c	low	full	two-part	?		3
Maidanetske	Mai-8	A	T-C1		1	1	2	334.0	23.0	14.5	1.6	2a	low	empty	one part	yes		3
Maidanetske	Mai-9	A	T-C1			1	1	135.0	15.0	9.0	1.7	2b	low	empty	one/two-part?	yes		6
Maidanetske	Mai-10	A	T-C1			1	1	162.0	18.0	9.0	2.0	3	low	empty	one/two-part?	yes?		6
Maidanetske	Mai-11	A	T-C1			1	1	158.0	17.0	9.3	1.8	3	low	empty	one part	yes		6
Maidanetske	Mai-12	A	T-C1			1	1	258.5	23.5	11.0	2.1	2b	low	empty	one part	yes		4
Maidanetske	Mai-13	A	T-C1			1	1	122.5	17.5	7.0	2.5	2c	low	empty	one part	?		4
Moshuriv 1	Mos-1	A	T-C1	1	1		2	238.7	31.0	7.7	4.0	8b	high	full	two-part	?	ENE	2
Moshuriv 1	Mos-2	A	T-C1		1	1	2	134.6	15.3	8.8	1.7	2a	low	empty	one part	?		3
Moshuriv 1	Mos-3	A	T-C1		1	1	2	149.2	16.4	9.1	1.8	2b	low	empty	one part	yes		3
Moshuriv 1	Mos-4	A	T-C1		1	1	2	96.4	14.6	6.6	2.2	2a	low	empty	one part	?		3
Nebelivka	Neb-1	A	T-B2	1	1	1	3	1200.0	60.0	20.0	3.0	7	high	partly empty	three-part	yes	ENE	2
Nebelivka	Neb-2	A	T-B2	1	1	1	3	375.0	25.0	15.0	1.7	2b	low	empty	one part	no		3
Nebelivka	Neb-3	A	T-B2	1	1	1	3	551.3	31.5	17.5	1.8	3	low	full	?	?		3
Nebelivka	Neb-4	A	T-B2		1		1		>10.0	16.5		2	low	empty	?	no		3
Nebelivka	Neb-5	A	T-B2		1	1	2	173.3	16.5	10.5	1.6	2b	low	empty	one part	yes		3
Nebelivka	Neb-6	A	T-B2	1	1	1	3	240.5	18.5	13.0	1.4	2	low	?	one part	?		3
Nebelivka	Neb-7	A	T-B2	1	1	1	3	433.5	25.5	17.0	1.5	2b	low	empty	one part	yes		3
Nebelivka	Neb-8	A	T-B2	1	1	1	3	260.0	20.0	13.0	1.5	3	low	empty	one part	yes		3
Nebelivka	Neb-9	A	T-B2	1	1	1	3	527.3	28.5	18.5	1.5	2b	low	empty	one part	yes?		3
Nebelivka	Neb-10	A	T-B2	1	1	1	3	376.0	23.5	16.0	1.5	2b	low	empty	one part	yes		3
Nebelivka	Neb-11	A	T-B2	1	1	1	3	336.0	21.0	16.0	1.3	2b	low	empty	one part	yes		3
Nebelivka	Neb-12	A	T-B2	1	1	1	3	387.5	25.0	15.5	1.6	2b	low	empty	one part	yes		3
Nebelivka	Neb-13	A	T-B2	1	1	1	3	408.0	24.0	17.0	1.4	5a	low	partly empty	?	?		3
Nebelivka	Neb-14	A	T-B2	1	1	1	3	345.0	23.0	15.0	1.5	2b	low	?	one part	?		3
Nebelivka	Neb-15	A	T-B2			1	1	204.8	19.5	10.5	1.9	2a	low	empty	one part	no		5
Nebelivka	Neb-16	A	T-B2			1	1	268.8	21.5	12.5	1.7	2a	low	empty	one part	no		5
Nebelivka	Neb-17	A	T-B2			1	1	170.5	15.5	11.0	1.4	2	low		?			5
Nebelivka	Neb-18	A	T-B2	1			1	340.0	34.0	10.0	3.4	5b	low	full	?	?		5
Nebelivka	Neb-19	A	T-B2			1	1	204.0	17.0	12.0	1.4	2a	low	empty	one part	no		5
Nebelivka	Neb-20	A	T-B2			1	1	228.0	19.0	12.0	1.6	3	low	?	?	?		5
Petreni	Pet-1	C	T-B2-C1	1	1	1	3	368.0	32.0	11.5	2.8	8b	high	full	two-part	?		2
Petreni	Pet-2	C	T-B2-C1		1		1	133.0	14.0	9.5	1.5	5a	low	full	?	?		3
Petreni	Pet-3	C	T-B2-C1		1		1	131.3	15.5	8.5	1.8	5a	low	full	?	?		3
Petreni	Pet-4	C	T-B2-C1		1		1	>140.0	>12.5	11.2		5a	low	full	?	?		3
Petreni	Pet-5	C	T-B2-C1		1		1	150.0	16.3	9.2	1.8	5a	low	full	?	?		3
Petreni	Pet-6	C	T-B2-C1		1		1	182.3	13.5	13.5	1.0	1	low	empty	one part	?		3
Petreni	Pet-7	C	T-B2-C1		1		1	164.7	13.5	12.2	1.1	1	low	empty	one part	?		3
Petreni	Pet-8	C	T-B2-C1		1		1	201.0	20.2	10.0	2.0	5a	low	full	?	?		3
Petreni	Pet-9	C	T-B2-C1	1	1		2	168.4	18.3	9.2	2.0	5a	low	full	?	?		4
Petreni	Pet-10	C	T-B2-C1	1	1		2	148.7	17.7	8.4	2.1	5a	low	full	?	?		4
Petreni	Pet-11	C	T-B2-C1	1	1		2	184.8	21.0	8.8	2.4	5a	low	full	?	?		4
Petreni	Pet-12	C	T-B2-C1	1	1		2	162.7	16.6	9.8	1.7	5a	low	full	?	?		4
Petreni	Pet-13	C	T-B2-C1	1	1		2	137.3	15.6	8.8	1.8	5a	low	full	?	?		4
Stolniceni	Sto-1	C	T-B2	1	1	1	3	254.0	28.5	9.0	3.2	8b	high	full	two-part	?	NE	2
Stolniceni	Sto-2	C	T-B2	1	1	1	3	185.5	23.0	8.8	2.6	8b	high	full	two-part	?	NE	2
Talianky	Tal-1	A	T-C1 late		1	1	2	144.0	18.0	8.0	2.3	2c	low	empty	one part			4
Talianky	Tal-2	A	T-C1 late		1		1	161.0	22.0	7.2	3.1	8a	low	partly empty	two-part	yes?		6
Talianky	Tal-3	A	T-C1 late				0	176.0	25.0	8.0	3.1	8b	dwelling	full	two-part	?		4
Talianky	Tal-4	A	T-C1 late		1	1	2	101.0	16.6	6.2	2.7	2c	low	empty	one part	yes		4
Talianky	Tal-5	A	T-C1 late		1	1	2	93.0	15.2	6.0	2.5	2c	low	partly empty	one part	yes?		4
Talianky	Tal-6	A	T-C1 late		1	1	2	207.0	22.0	9.8	2.2	2c	low	partly empty	one part	?		4
Talianky	Tal-7	A	T-C1 late		1	1	2	195.0	22.0	8.8	2.5	2c	low	partly empty	one part	yes?		4
Talianky	Tal-8	A	T-C1 late		1	1	2	176.0	20.5	8.7	2.4	2c	low	empty	one part	yes		4
Talianky	Tal-9	A	T-C1 late		1	1	2	118.0	15.0	7.7	1.9	2c	low	empty	one part	yes		4
Talianky	Tal-10	A	T-C1 late		1	1	2	105.0	15.0	7.0	2.1	2c	low	partly empty	one part	yes?		4
Ternivka	Ter-1	B	T-B1-B2	1	1		2	317.3	33.4	9.5	3.5	5b	high	full	?	?	ESE	2
Trostianchyk	Tro-1	B	T-B2	1			1	175.0	23.0	7.6	3.0	?	dwelling?	?	?			0
Trostianchyk	Tro-2	B	T-B2	1	1		2	195.0	22.4	8.7	2.6	5b	high	full	?	yes?	NNE	2
Veselyi Kut	Ves-1	A	T-B1-B2		1	1	2	202.0	18.5	11.0	1.7	4	high	full	multi-part		SEE	2
Veselyi Kut	Ves-2	A	T-B1-B2			1	1	83.0	14.1	6.0	2.4	2c	dwelling?	?empty	one part			0
Veselyi Kut	Ves-3	A	T-B1-B2			1	1	105.0	17.9	6.0	3.0	2	dwelling	empty	?			4
Veselyi Kut	Ves-4	A	T-B1-B2		1	1	2	94.0	13.5	7.0	1.9	2b	low	empty	one part	yes		6
Veselyi Kut	Ves-5	A	T-B1-B2			1	1	66.0	11.0	6.0	1.8	2b	dwelling?	empty	one part	yes?		0
Veselyi Kut	Ves-6	A	T-B1-B2			1	1	98.0	13.5	7.0	1.9	2b	dwelling?	empty	one part	yes?		0
Viitivka	Vit-1	B	T-C1	1	1		2	352.8	39.2	9.0	4.4	8a	high	full	two-part	?	NE	2
Viitivka	Vit-2	B	T-C1	1	1		2	684.3	59.5	11.5	5.2	8b	high	full	two-part	?	NE	2
Viitivka	Vit-3	B	T-C1	1	1		2	310.3	36.5	8.5	4.3	9	high	partly empty	two-part	?	NE	2
Vilshanka	Vil-1	B	T-B1-B2	1	1		2	389.0	37.4	10.4	3.6	8b	high	full	two-part	?	ENE	2
Volodymyrivka	Vol-1	A	T-B2	1	1	1	3	>443.0	>50.8	23.5		7?	high	empty	three-part	?	NE	2
Volodymyrivka	Vol-2	A	T-B2	1	1	1	3	532.0	28.0	19.0	1.5	2a	low	empty	one part	no		3
Volodymyrivka	Vol-3	A	T-B2	1	1	1	3	356.5	23.0	15.5	1.5	2a	low	empty	one part	no		3
Volodymyrivka	Vol-4	A	T-B2	1	1	1	3	387.5	25.0	15.5	1.6	2a	low	empty	one part	no		3
Volodymyrivka	Vol-5	A	T-B2	1	1	1	3	609.0	29.0	21.0	1.4	2a	low	empty	one part	yes		3
Volodymyrivka	Vol-6	A	T-B2	1	1	1	3					2a	low	empty	one part	no		3
Volodymyrivka	Vol-7	A	T-B2	1	1	1	3	555.0	30.0	18.5	1.6	3	low	empty	one/two-part?	?		3
Volodymyrivka	Vol-8	A	T-B2	1	1	1	3	>187.0	>12.5	15.0		2b	low	empty	?	yes		3
Volodymyrivka	Vol-9	A	T-B2	1	1	1	3	353.6	26.0	13.6	1.9	2a	low	empty	one/two-part?	?		6
Volodymyrivka	Vol-10	A	T-B2	1		1	2	220.8	18.4	12.0	1.5	2b	low	empty	one part	yes		5
Volodymyrivka	Vol-11	A	T-B2	1		1	2	234.0	18.0	13.0	1.4	3	low	partly empty	one part	?		5
Zabolotne	Zab-1	B	T-B1-B2	1	1	1	3	912.0	57.0	16.0	3.6	9	high	partly empty	two-part	?	NE	2
Zabolotne	Zab-2	B	T-B1-B2		1	1	2	305.0	30.5	10.0	3.1	6b	high	partly empty	two-part	?	NE	2

### Methods

#### Reconstructing the appearance of a mega-structure

The evaluation of the architectural remains, distribution patterns of daub, and negative imprints of timbers allows the reconstruction of the general building design of the investigated mega-structure 3. This evaluation was based on the field documentation which included excavations plans, photos, and the recording of location and masses of daub and movable finds either with point coordinates or in a grid of 1 x 1 m cell size. During excavation, daub was systematically documented to reconstruct both architectural attributes and wood resources [37, 45, 46]. This documentation included the mapping of daub fragments and a typological classification of daub pieces based on their material attributes and on the direction and dimension of imprints of structural (wooden) elements. To determine the architectural construction, the location of wood imprints was mapped differentiated according to the diameter of logs and the width of split wood planks.

Mapping of artefact distributions was performed in order to identify activity zones within the excavated mega-structure 3. Refitting of ceramic vessels and mapping of pottery fragmentation were used for the consideration of taphonomic formation processes and interpretations of primary and secondary artefact distributions.

#### Comparison of artefact and botanical macro-remain assemblages of mega-structures and dwellings

Qualities and quantities of objects which were associated with the mega-structure and two normal dwellings provide an estimate for reconstructing activities performed related to these different objects. Generally, similar formation processes of find assemblages are assumed for the compared different types of buildings. Specifically, we assume that possible behavioural differences during abandonment of the houses and the mega-structures are balanced through the inclusion of materials from probably waste disposal areas in the surrounding of the buildings. Additionally, the degree of fragmentation of pottery is used to evaluate the questions if vessels were found in primary or secondary waste disposal contexts.

The inventory comparisons take into account the absolute amount of objects and partly the density of objects per cubic metre of excavated soil. Thus, e.g. the frequency of particular vessel classes allows conclusions about certain functional aspects of a particular find context. This is especially the case for vessels associated with storage purposes and for food consumption.

Vessels were quantified as follows: After intensive processing and refitting of vessel units, the identification and counting of individual vessels units took place in consideration of typological differences concerning size, morphology, and decorations. A minimal number of vessel units (MNI) was determined by recording the preserved percentages of rims, bellies, and bottoms and setting a complete vessel as 100%. An alternative quantification of vessel classes is based on the count of recorded vessel units, reflected in the number of data sets in the project database. The percentage of sherds with engobe on their inner surface provides a relative measure for the percentage of bowls in an assemblage.

For botanical macro-remains 214 samples of sediment of 10 litres each were taken in the trench 111 (198 of these samples come from the stratigraphy of the mega-structure 3). Systematic sampling was carried out grid-wise and intensified for every second quadrant (1 m^2^) for the ancient soil surface. Flotation has been carried out on these samples, using a metal sieve of 300 μm, as previously done for the other samples from the site [45, 47]. Overall 414 charred botanical finds have been retrieved from trench 111, but preservation conditions allowed the taxonomic identification of only 305 of those. The carbonised remains found in trench 111 (S1 Table) include cereal grains (*Triticum* sp., *Hordeum* sp., mostly cereals indet.), pulses (Fabaceae) and feather-grass awns (*Stipa* sp.).

#### Calculation of the use group size

The use group sizes of mega-structures were quantified based on the numerical ratio between mega-structures and dwellings. Therefore, the key-value of the estimates is the determination of the number of dwellings that belonged in purely arithmetical terms to a single mega-structure. Within settlements, we assume the rough contemporaneity all low-level mega-structures visible in the magnetic plans, since this is clearly indicated by the uniform distribution of the buildings. The population estimates are based on the average floor space of 7 m^2^ per person determined using a cross-cultural ethnographic data-set [48]. For each site the average size of residential buildings was taken into account. We are aware that the values calculated in this schematic way of quantification provide only maximum use group sizes since the contemporaneity of the houses is assumed in the most cases. Nevertheless, the calculations are suitable for identifying regional and chronological trends regarding the size of use groups.

#### Detecting temporal and regional variability

Key parameters of the regional sample of mega-structures such as frequency, architectural design, positioning in settlements, and dimensions were analysed with regard to their variability in space and time. For mega-structure 3 from Maidanetske, the basis for the chronological investigations are ^14^C data calibrated and analysed using the software OxCal v. 4.3.2 [49, 50]. In the case of the regional data set, ^14^C calibrations of already established Tripolye periodization schemes were used [51, 52].

## Results

### What do mega-structures look like and differentiates them from domestic dwellings?

In the following section, the architectural design and the internal organization of an average mega-structure in Maidanetske will be evaluated. The extent to which the architecture and spatial organization of this building differs from normal houses is then examined by comparison with houses 44 and 59 from Maidanetske [34, 36, 37]. The extent to which the findings made for Maidanetske mega-structure 3 are representative is finally evaluated on the basis of comparisons with mega-structures in magnetic plans and other archaeologically examined buildings.

#### Investigating a mega-structure: The case of Maidanetske mega-structure 3

Mega-structure 3 in trench 111 was chosen for excavation because the magnetic plan displayed both highly magnetised and low magnetised sections within a clearly demarcated area. The different parts were hypothetically interpreted as roofed and not-roofed areas for different activities. During the excavation it turned out that the area was used for settlement activities already before the construction of the mega-structure.

In order to determine the absolute chronology, eleven bone-samples were ^14^C dated by accelerator mass spectroscopy at the Poznan Radiocarbon Laboratory from different stratigraphic contexts of trench 111 (Table 3). Pre-mega-structure activities are dated through the dates Poz-87599–Poz-87605 deriving from pits and a levelling layer below the floor of the mega-structure. The phase of use of mega-structure 3 is represented by two dates from disarticulated bones which were found in-between the wall debris of the mega-structure (Poz-87598, Poz-87610). Post-mega-structure activities are represented by two dates from the layer directly above the wall debris (Poz-87609, Poz-87721).

**Table 3:** Maidanetske, list of ^14^C dates from trench 111.

laboratory-id	14C age (BP)	N (%)	C (%)	col (%)	find-id	feature-id	level	grid x	grid y	material	taxon	context
Poz-87721	4900 ± 40	0.9	7.0	1.0	1110275	111002	2	F	9	bone	bos	layer above mega-structure,
Poz-87609	5055 ± 35	2.5	10.4	5.6	1110085	111002	2	L	5	bone	bos	layer above mega-structure
Poz-87610	5035 ± 35	2.5	10.9	4.4	1110689	111003	3	F	9	bone	sus	wall debris of mega-structure
Poz-87598	4990 ± 35	2.9	11.0	5.9	1110750	111003	3	M	14	bone	bos	wall debris of mega-structure
Poz-87599	5010 ± 35	4.5	14.5	3.0	1111565	111025	4a	J	13	bone	bos	cultural layer below mega-structure
Poz-87600	4970 ± 30	2.9	11.0	2.0	1110981	111025	3	L	9	bone	bos	cultural layer below mega-structure
Poz-87601	5020 ± 35	1.8	9.7	2.4	1111294	111026	4e	K	9	bone	bos	upper edge of pit 111/1
Poz-87602	4955 ± 30	1.2	7.9	1.1	1111077	111026	4e	K	9	bone	bos	upper edge of pit 111/1
Poz-87603	4990 ± 35	4.3	13.6	8.2	1111368	111029	4d	J	5	bone	bos	pit 111/3 below mega-structure
Poz-0	>0	0.3	5.7		1111373	111029	4d	K	5	bone	bos	pit 111/3 below mega-structure
Poz-87604	5000 ± 35	2.4	9.5	3.1	1111542	111033	profile 30	F	6	bone	bos	lower level of pit 111/2
Poz-87605	5035 ± 35	2.7	10.9	4.2	1111519	111032/33	profile 30	F	8	bone	bos	lower level of pit 111/2

**Fig 3:**
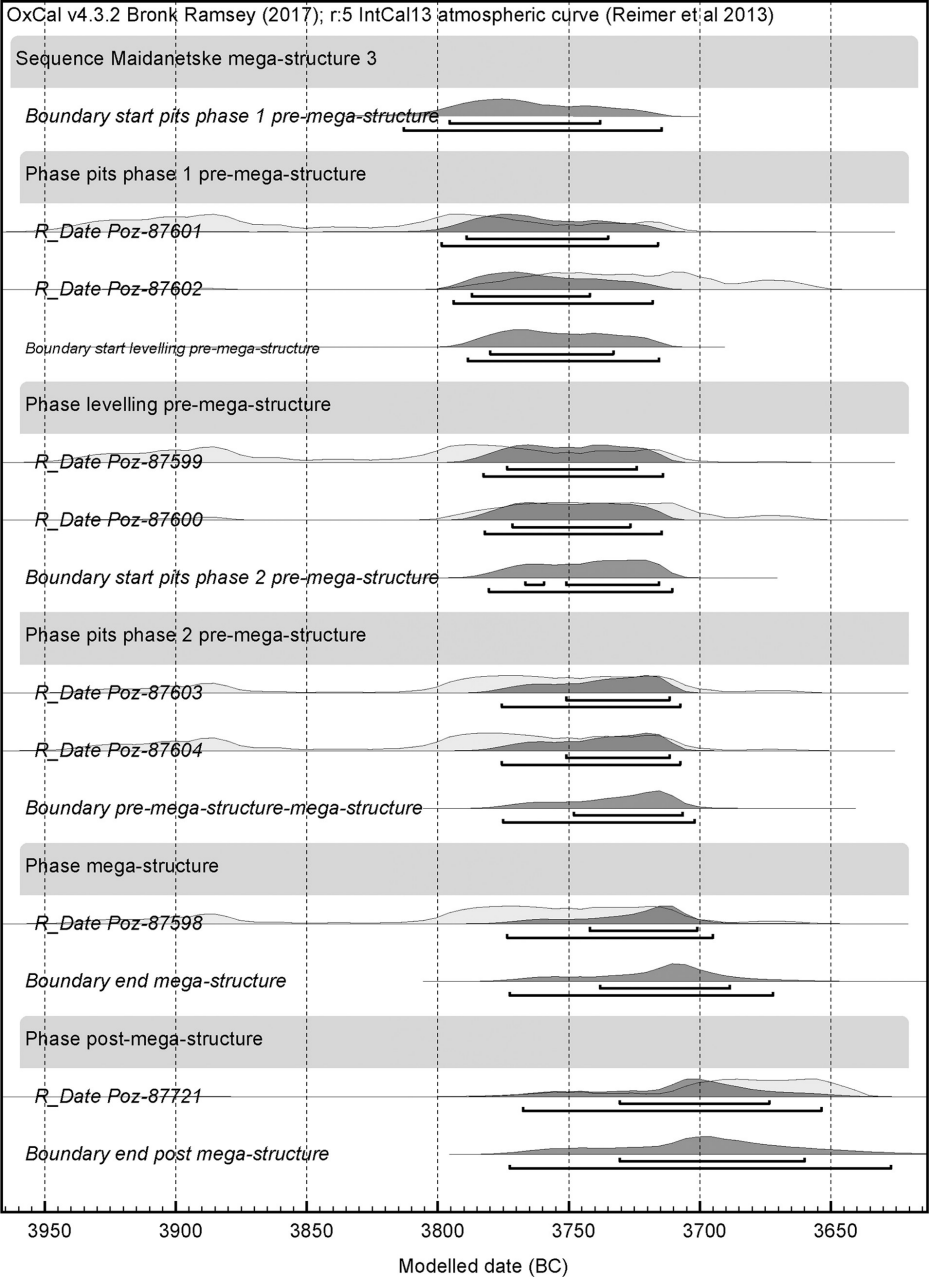
Maidanetske. Bayesian models of ^14^C dates from trench 111 plotted on the calibration curve.

Overall, the dates fall into a plateau of the calibration curve and the following steep section, covering a long range of about 300 years between 3950 and 3650 BCE. Through application of Bayesian modelling and the use of the function *boundary* with the assumption of two successive occupation phases and several events, the range of the dates becomes significantly narrowed, roughly into the 38^th^ century BCE (Fig 3 and S1 File). However, the overall probability of this model amounts to only 40% (A_model_ = 33.8) due to widely identical dates from the different phases. Higher overall probabilities of more than 100% can only be obtained through exclusion of the potential (too old) outliers Poz-87605, Poz-87609, and Poz-87610. The dating results imply that mega-structure 3 was constructed during phase 3 of the site chronology suggested by Ohlrau [31]. Consequently, mega-structure3 from Maidanetske was build related to the rapid population increase of the 38th century and abandoned at the beginning of phase 4, related to the starting population decrease.

**Fig 4:**
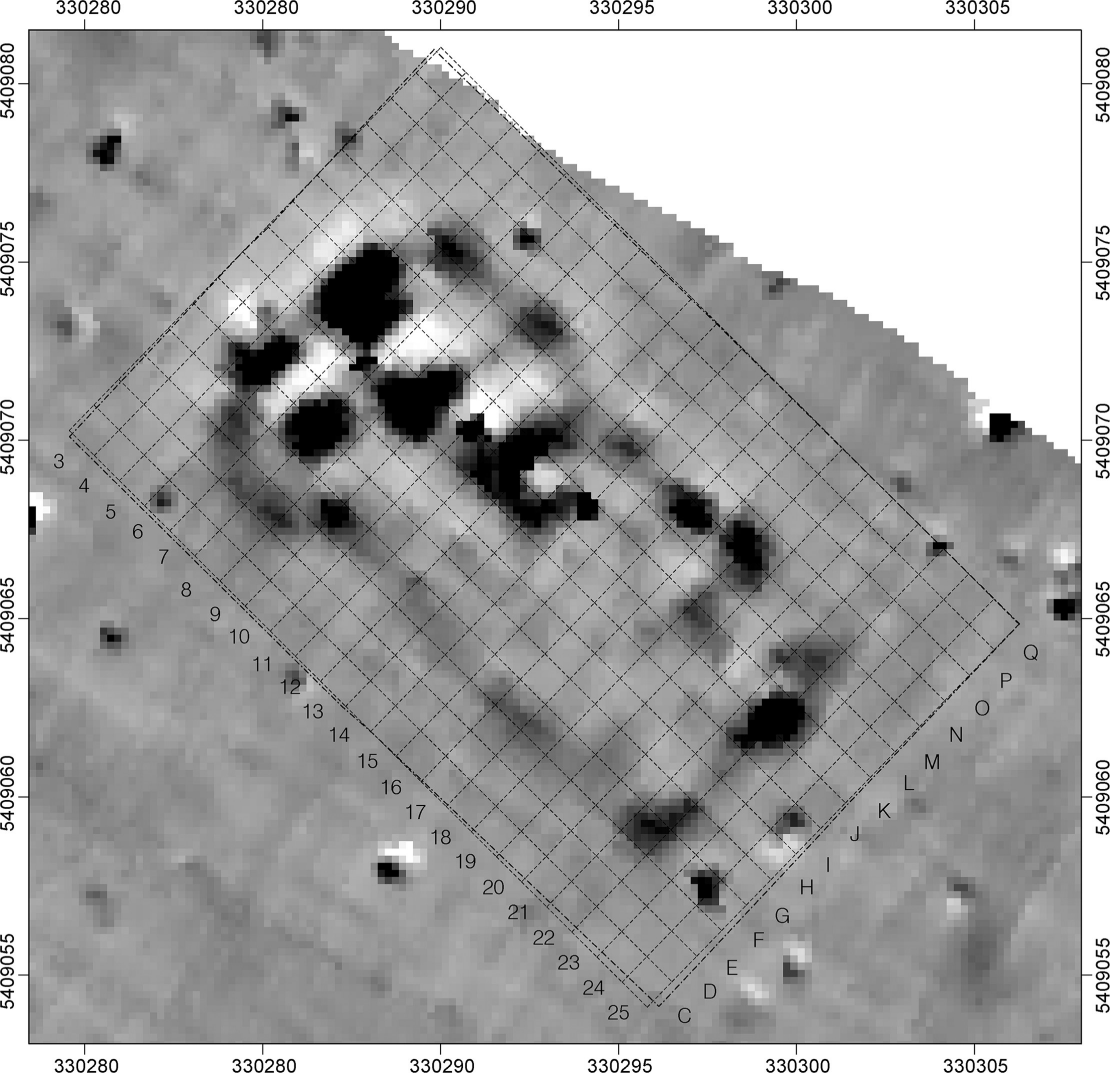
Maidanetske, trench 111, section of the magnetic plans with anomalies of mega-structure 3.

In the magnetic plan of the Maidanetske settlement, the architectural remains of mega-structure 3 appeared as a northwest-southeast aligned anomaly with a floor size of approximately 190 m^2^ (dimensions 19 x 10 m) (Fig 4). Trench 111, opened over this anomaly, measured 23 x 15 m and the daub package of mega-structure 3 was encountered buried under a Chernozem 0.5 m thick.

**Fig 5:**
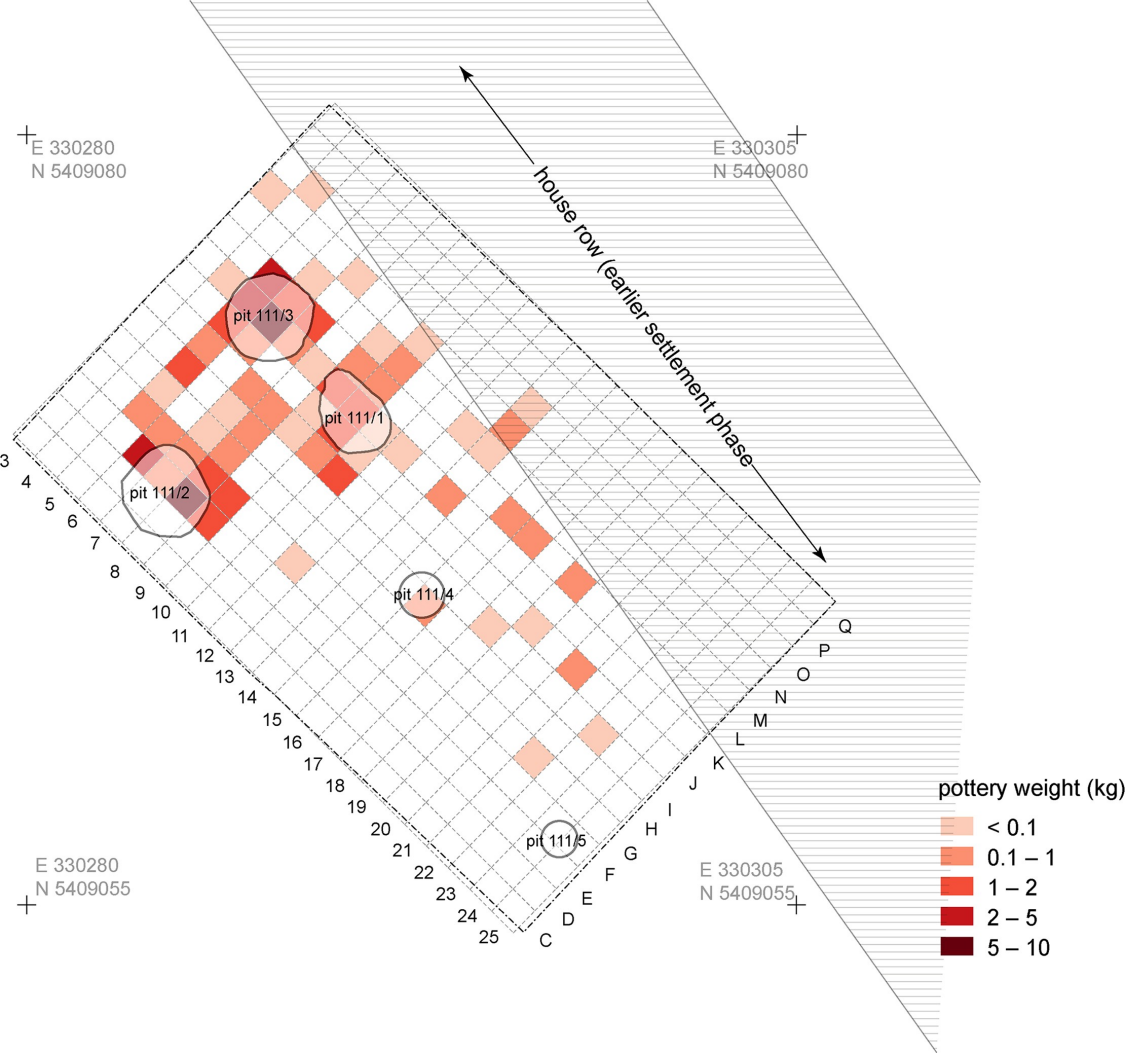
Maidanetske, features of the first building phase in trench 111 below mega-structure 3 with location of pits and a row of dwellings.

**Fig 6:**
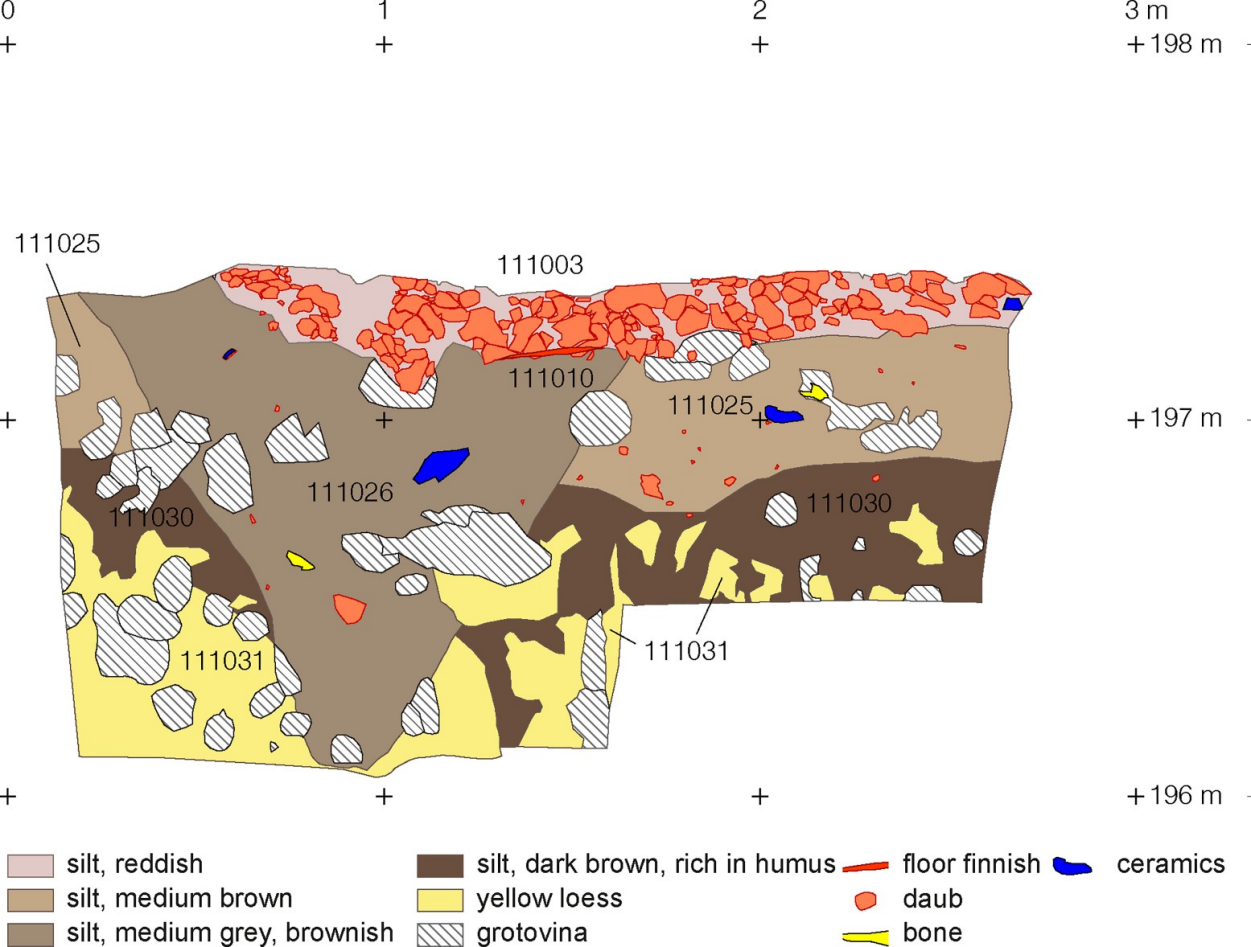
Maidanetske, trench 111, drawing of profile 32 through pit 111/3 below mega-structure 3 (location see Fig 7).

Mega-structure 3 was built in an area in which stratigraphic evidence indicates an earlier settlement phase. To this pre-mega-structure occupation belong the pit 111/1, perhaps the pits 111/2–111/5, and a massive levelling layer with numerous pottery finds (Figs 5 and 6). The pits were probably located in the back area of a northwest-southeast running row of houses whose position can be reconstructed based on remaining buildings in the magnetic plan. As the massive filling of pit 111/1 with daub located directly below the central fireplace of the mega-structure shows, the houses of the earlier phase were obviously burnt down and levelled to prepare the ground for the construction of the mega-structure.

**Fig 7:**
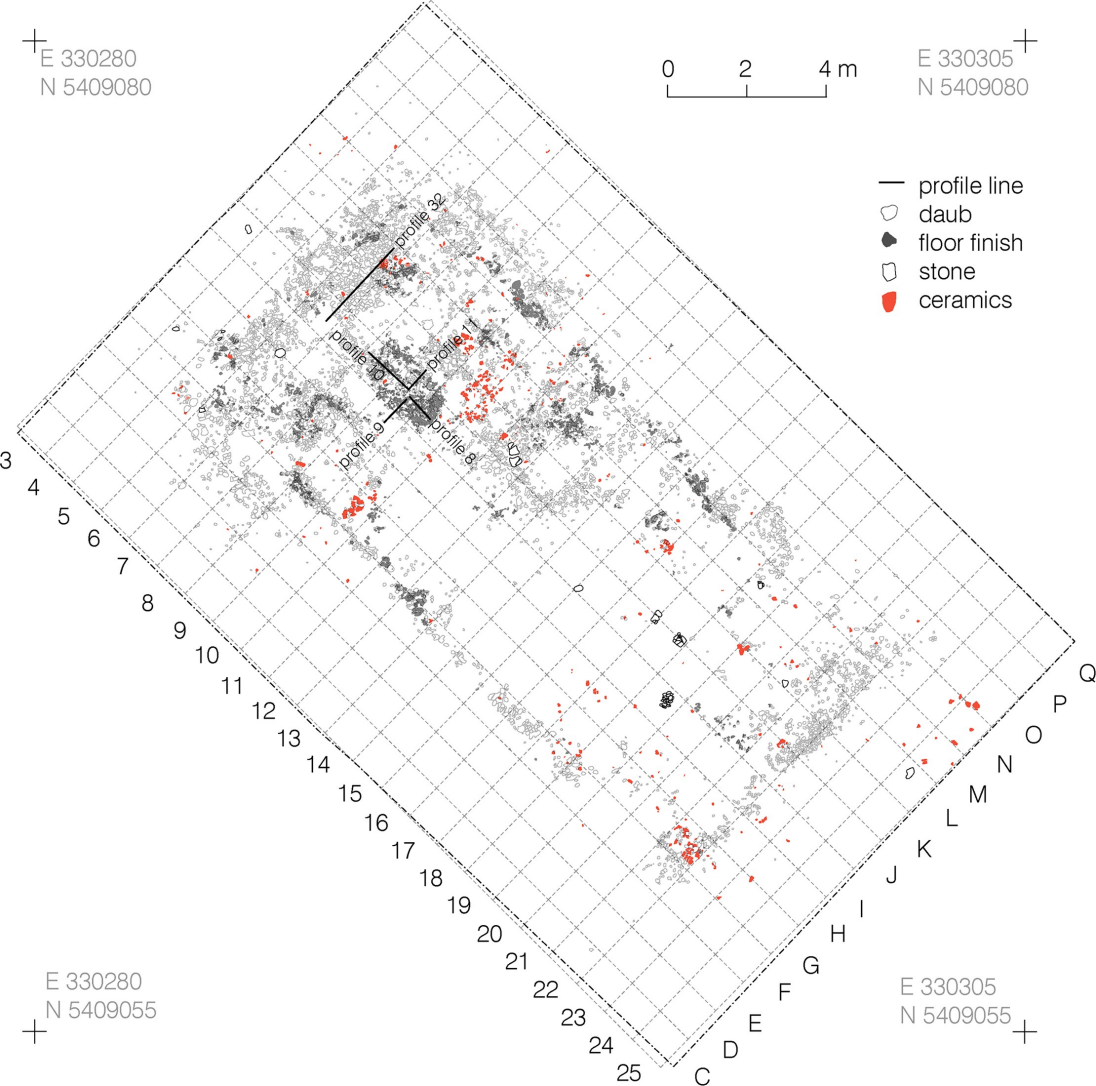
Maidanetske, mega-structure 3 in trench 111, drawing of daub from collapsed walls, floor, and pottery.

**Fig 8:**
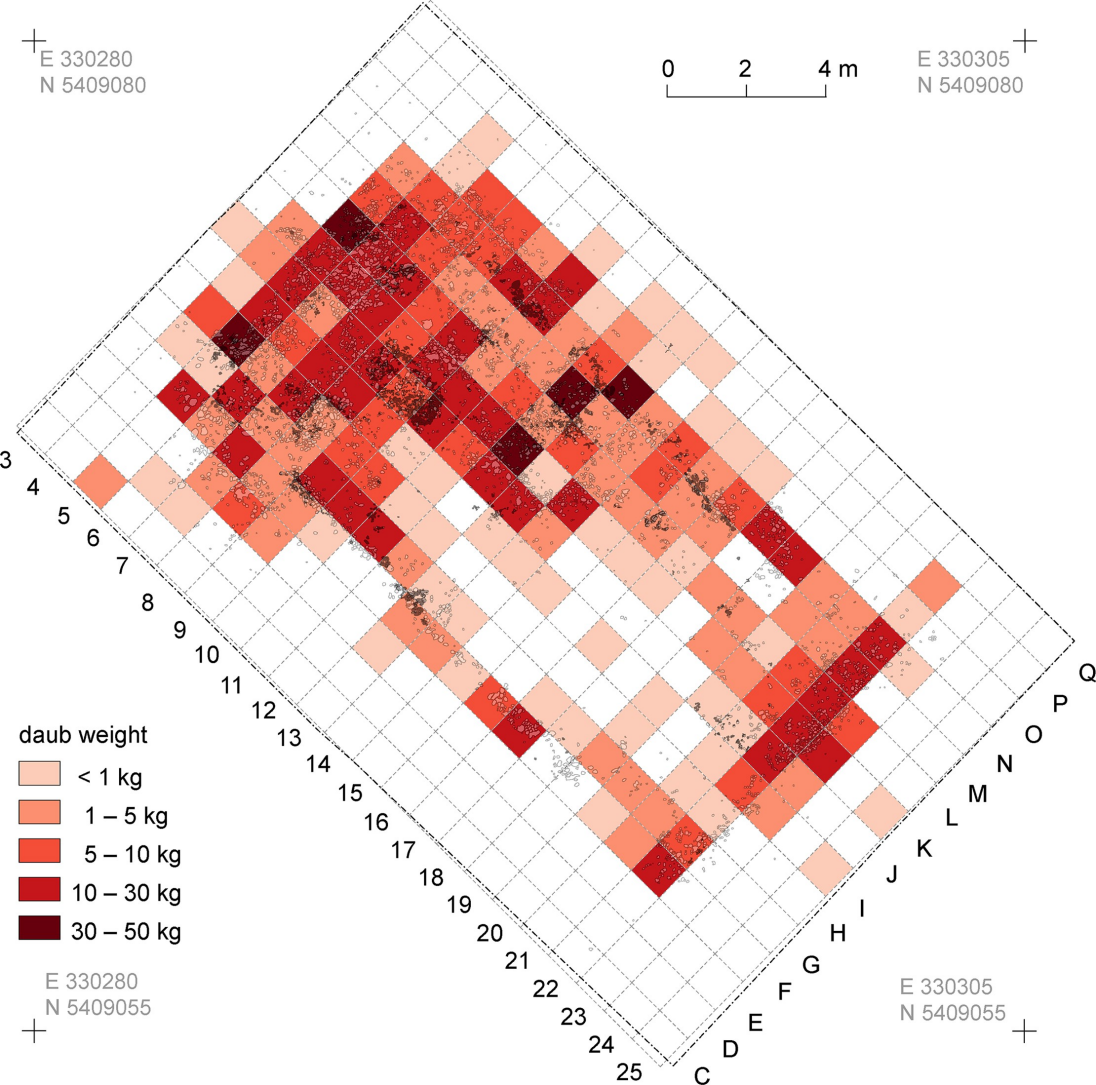
Maidanetske, weight of daub belonging to mega-structure 3 in trench 111.

Within the mega-structure, daub was not equally distributed, corresponding with the high and low magnetised areas visible in the magnetic plan (Figs 7 and 8) [46]. In some parts of the exterior walls and in the north-western half of the building, concentrations in quantities of between 10 and 50 kg/m^2^ were found. In contrast, a particularly low amount of daub in the range of up to 1 kg/m^2^ was documented in the southern quarter of the structure and the surrounding open space. In consequence, an internal division into northwestern and southeastern parts is clearly apparent.

**Fig 9:**
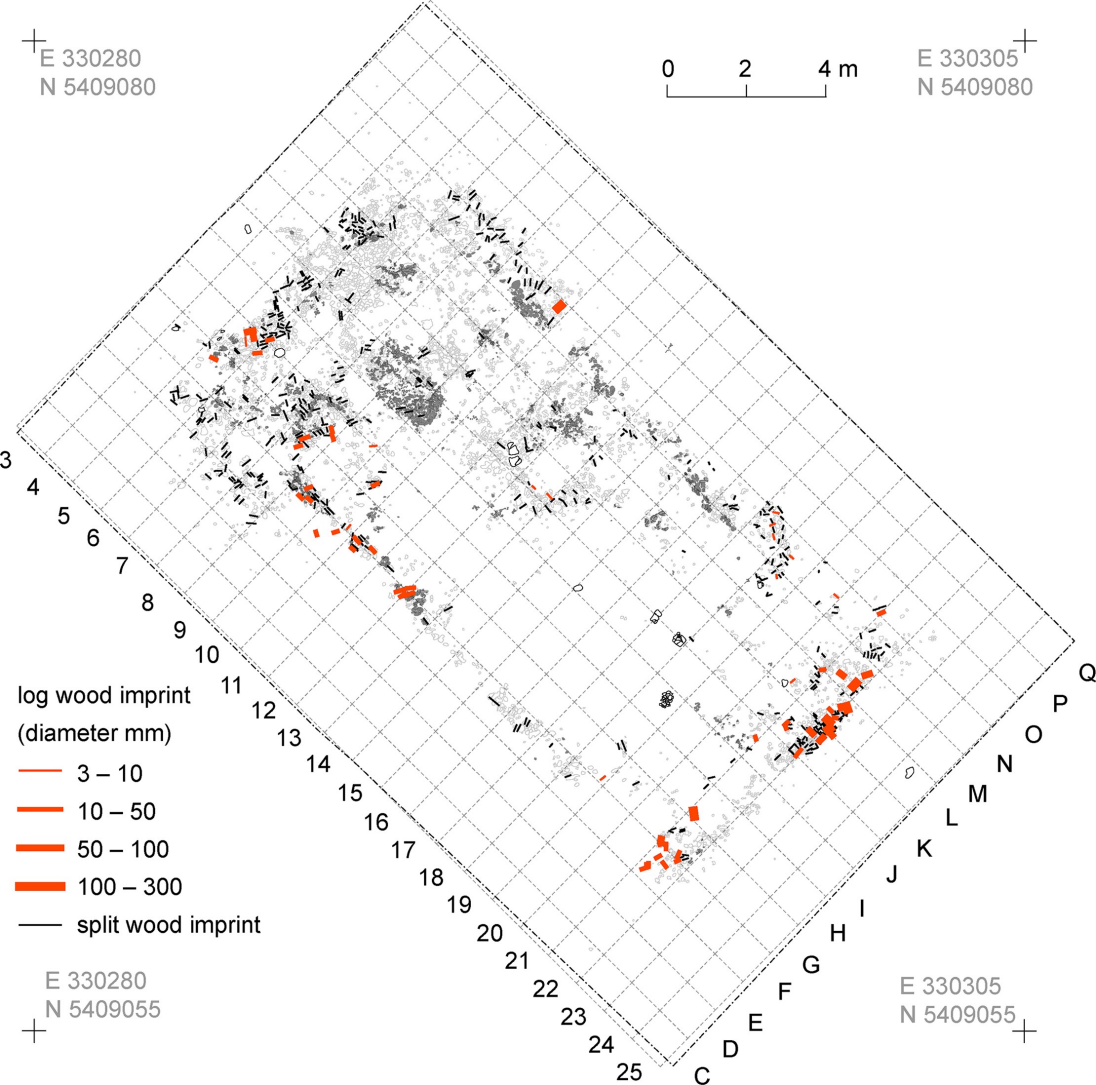
Maidanetske, kind, dimension, and direction of timber imprints from mega-structure 3.

The mega-structure was outlined by a lightweight outer wall made of clay-covered split wood and log timbers. Due to various post-depositional processes, this construction was preserved in different qualities. Based on analysis of negative imprints and the measurements of the burnt daub cover, the width of the wall is estimated to have been about 15–20 cm thick. As building timber, ash (*Fraxinus* 75%, n = 44) and oak (*Quercus* 19%, n = 11) were used whose dimensions generally fall below 10 cm [45]. Taking the wood imprints into account, both narrow sides and parts of the southwestern longitudinal part of the wall were constructed with log timbers, while the other parts of the wall were constructed mainly with split wood (Fig 9).

At the southern ends of the longitudinal walls daub-free areas about 1.4 m wide are interpreted as possible entrances. The southeastern narrow side of the mega-structure was particular massive, indicated by the largest diameters of log timbers. A daub concentration 7 m south of the northwestern narrow end might indicate the remains of an interior wall dividing the mega-structure in two parts. The internal wall probably reached 4 m across the house, but 3.50 m remained daub-free, perhaps as a passageway between the two parts of the structure. A small entrance about 1 m wide might also have existed directly north of the interior wall on the northeastern longitudinal side.

The orientation of the negative imprints in the split-wood suggests the timbers were aligned horizontally in the walls of the southeastern part and vertically in the walls of the northwestern part of the mega-structure. The lack of postholes could indicate a construction with horizontal beams as wall foundations. In the northwestern corner the daub remains with vertically oriented negative imprints might be remains of a gable wall which collapsed into the internal space of the mega-structure. The height of the original external wall can be reconstructed to about 3.5 m. Also daub remains of the internal wall suggest an original height of 3–3.5 m.

Below small-sized and chaff-tempered debris of the walls, remains of a burnt rammed earth floor were found in the entire area of the mega-structure (Fig 7). Since this mostly only poorly burnt and partly only 1 cm thick floor layer was preserved exclusively in those places which were also covered by wall debris, it was particularly badly preserved (due to low firing intensity) in the southeastern part of the structure. The floor under the debris of the exterior walls was in best condition and the floor layer was up to several centimetres thick. At the outer edge of the wall debris, even in the locations with better preservation, the floor layer suddenly stopped. Here, the floor layer was slightly raised upwards where it would have originally met the outer walls if they had been preserved in place. In consequence, it is suggested that all 190 m^2^ of the mega-structure’s interior were originally covered with a rammed earth floor. The outer edge of this rammed earth floor marked the position of the not preserved exterior walls.

In the northwestern part of the building different installations existed. Within the interior space only few remains of furnishings were recovered. However, spatial concentrations of a specific yellowish kind of daub in the northwestern part of the building might indicate destroyed furnishing elements. In normal dwellings such as house 44 similar material was used for the construction of bins and podiums [37].

**Fig 10:**
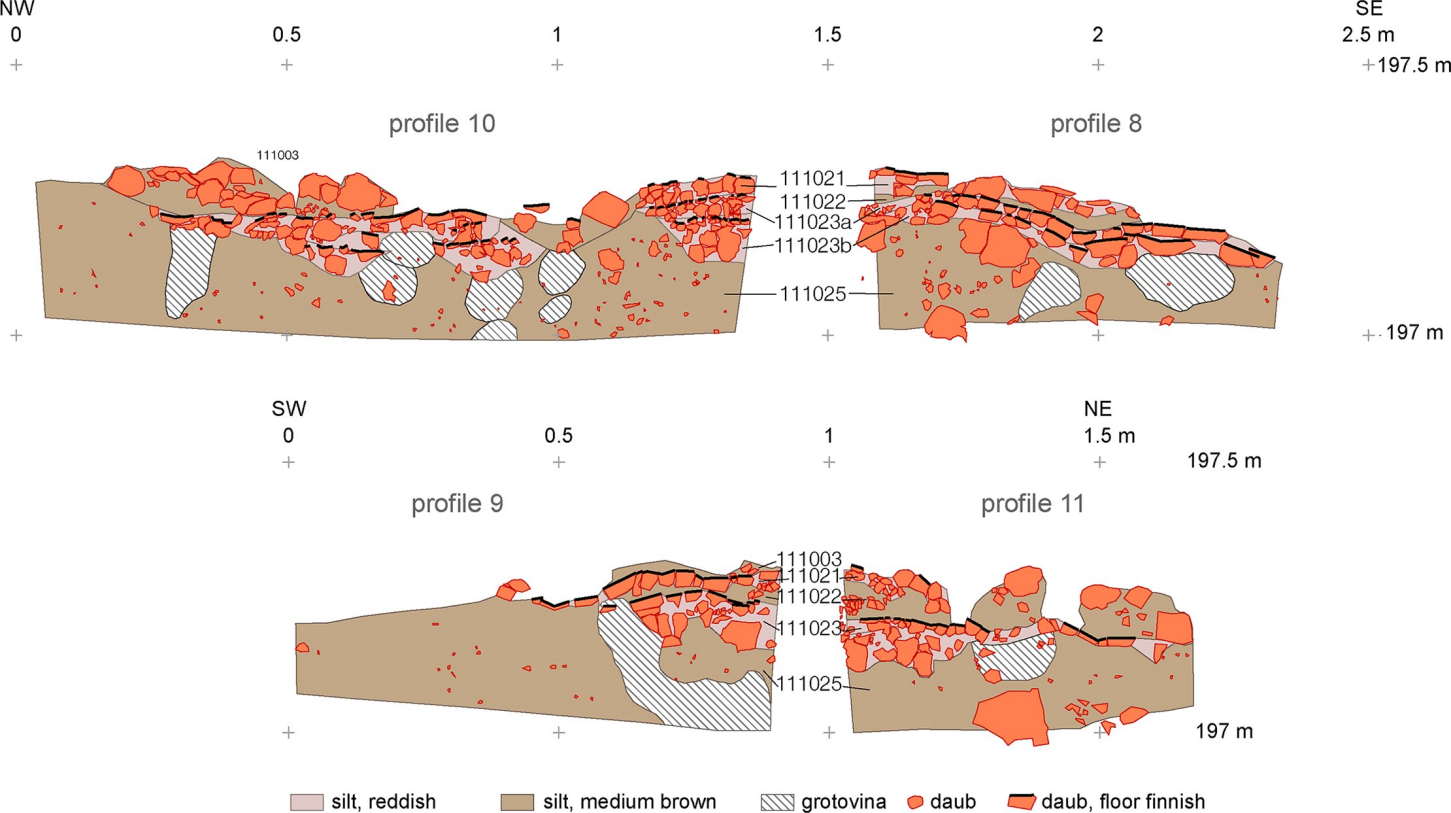
Maidanetske, profile sections through the fireplace of mega-structure 3.

An oval area 2.2 x 1.3 m, situated within the mega-structure three to five meters away from the northwestern narrow end along the longitudinal axis, marks a fireplace which was raised above the rest of the floor by several extra layers of tamped and burnt earth (I–J/8–10; Figs 7 and 10). Corresponding installations are a standard element of Tripolye houses [53]. Since they are sometimes decorated, they are frequently interpreted as altars [31, 53]. In the installation of mega-structure 3 from Maidanetske at least three successive screed layers lay one above another and testify to a longer-lasting use of the building. In contrast, no signs of floor renewals were determined in the remaining parts of the mega-structure.

The southeastern part of the mega-structure has a size of 10 x 7 m, measuring from the base of the interior wall, which collapsed probably in a southeastern direction. No archaeological features could be detected. In this respect, the southeastern part of the mega-structure is empty, but artefact distributions describe different activity zones.

**Fig 11:**
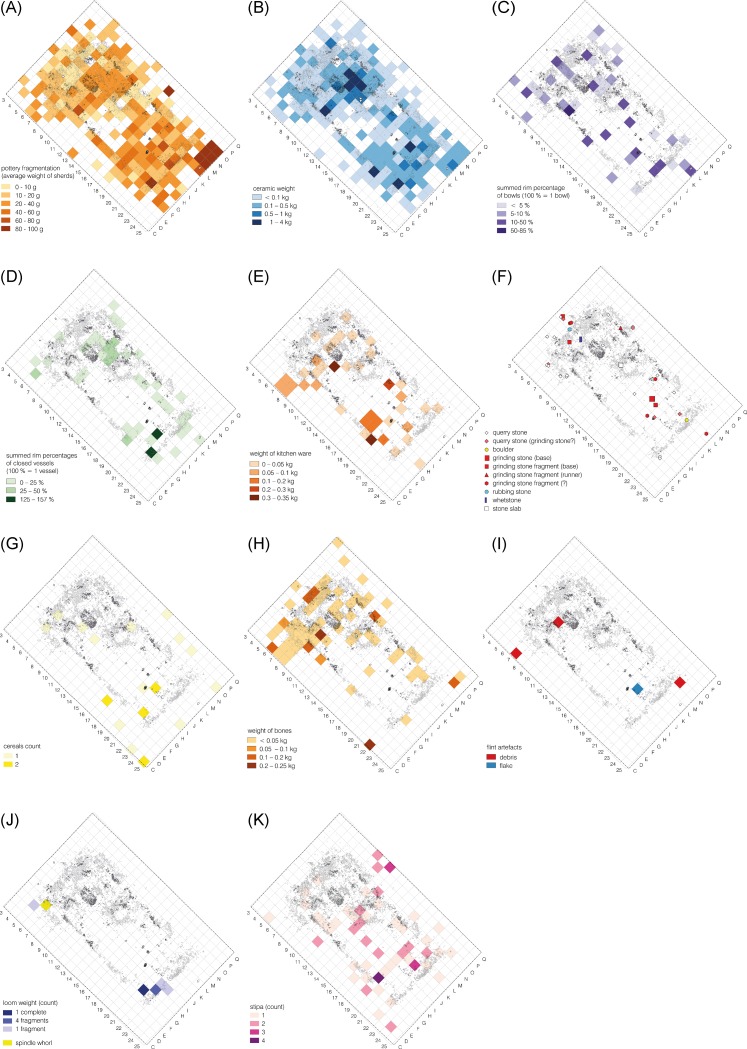
Maidanetske, find distributions in mega-structure 3. (A) Ceramic fragmentation. (B) Ceramics. (C) Bowls. (D) Closed/half-closed shapes. (E) Kitchen ware. (F) Ground stone artefacts. (G) Cereal Grains. (H) Animal bones. (I) Flint artefacts. (J) Remains of textile production. (K) *Stipa* seeds.

Artefact distribution patterns provide information about the depositional processes and activities which took place within the mega-structure 3. In the case of certain artefact categories such as querns, bones, and remains of textile production a distinction can be made between activities performed repeatedly over a longer period of time and actions done just before the abandonment of the mega-structure. On the other hand, due to the probable deliberate burning of the mega-structure usual abandonment processes such as the disposal of foreign waste occurred perhaps not or in lower scale. The find distributions of pottery are most likely determined by both, long and short term depositional processes. The overall low degree of fragmentation seems to indicate that pottery was fragmented during a primary context of use (Fig 11).

**Fig 12:**
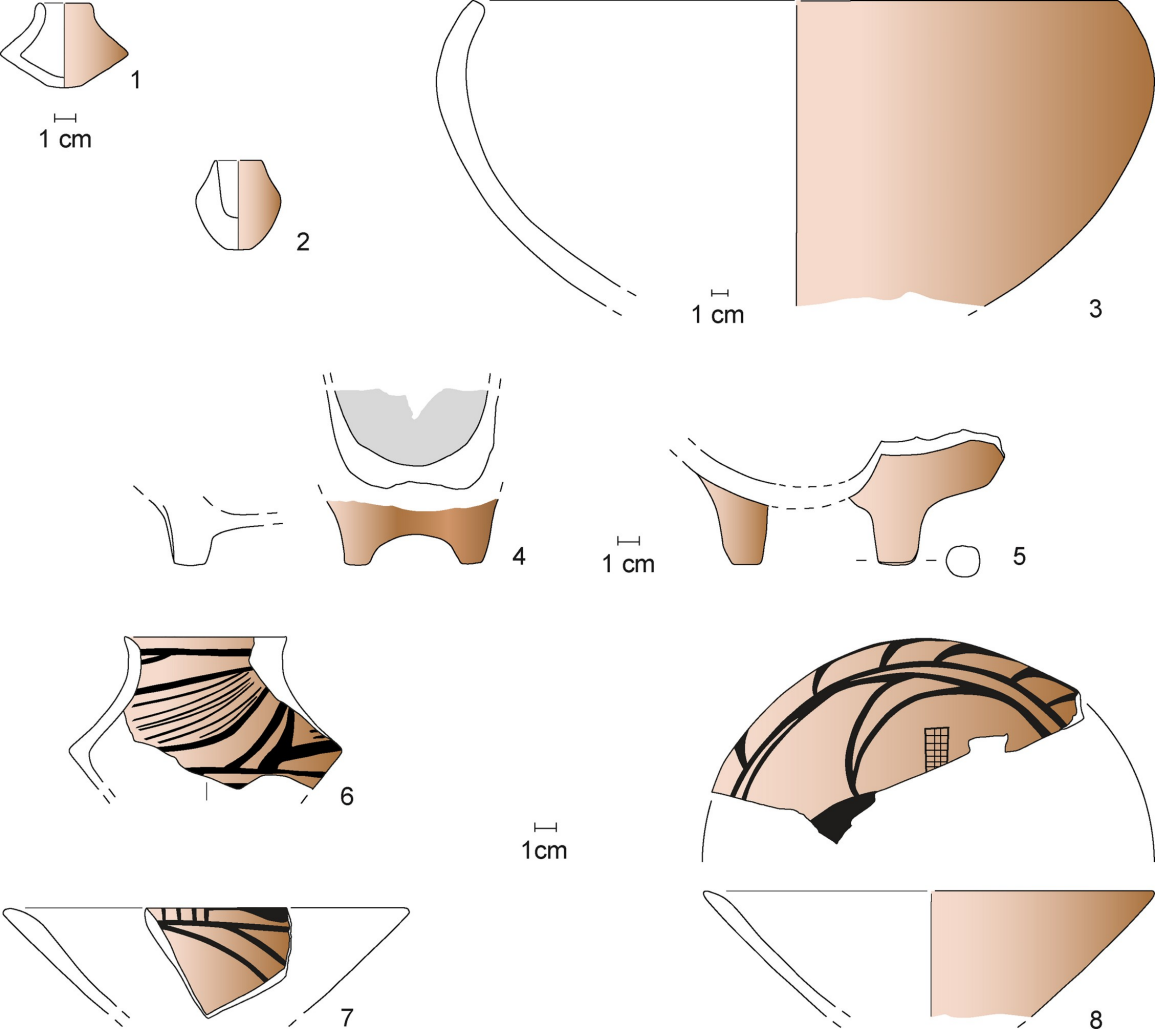
Maidanetske, ceramic inventory of mega-structure 3: Cup (1), bowls (3–5, 7, 8), miniature vessel (2), and goblet (6).

**Fig 13:**
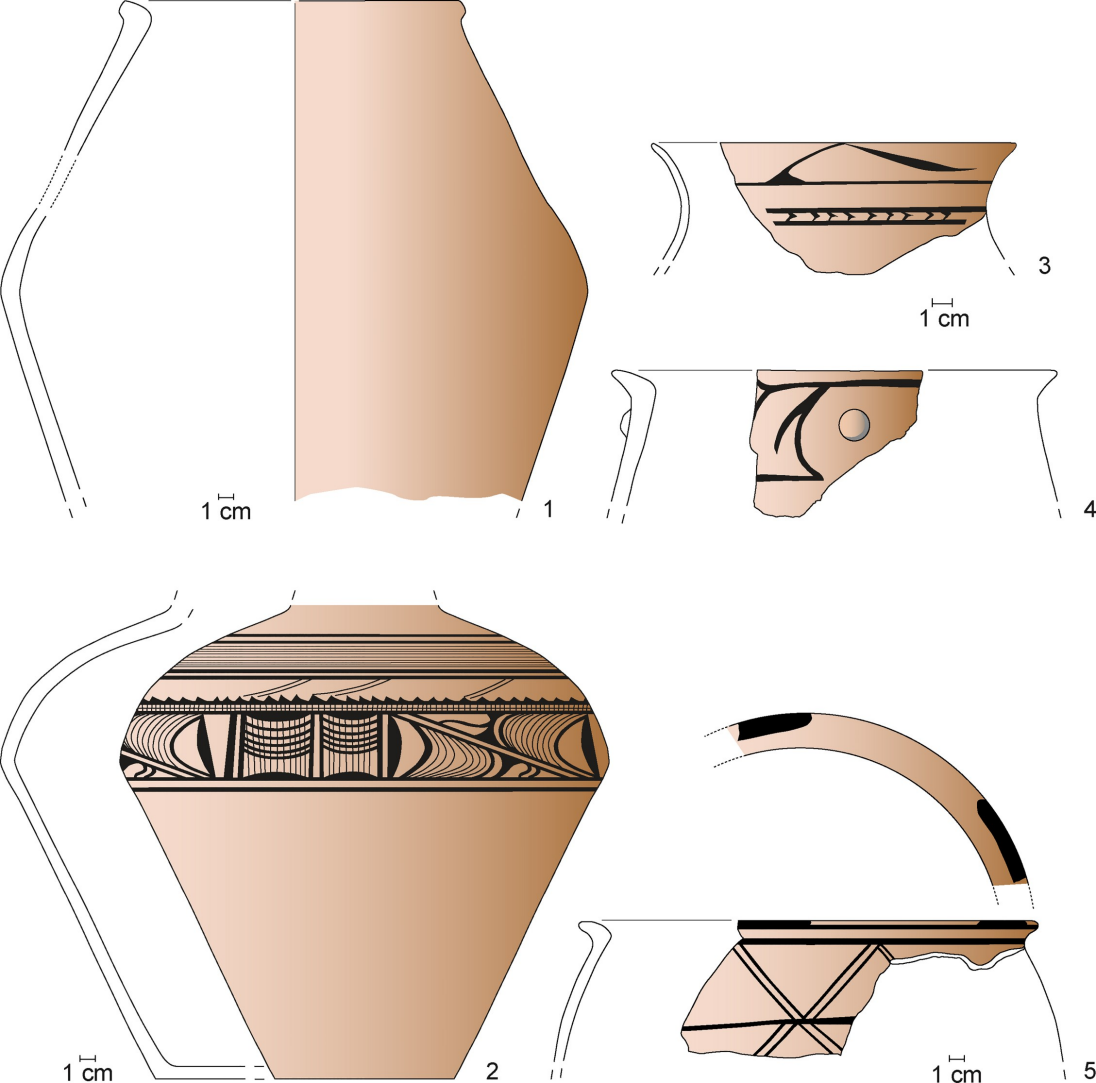
Maidanetske, ceramic inventory of mega-structure 3: Biconical vessel (1), crater (3), biconical/sphero-conical vessels (4, 5), and sphero-conical vessel (2).

**Fig 14:**
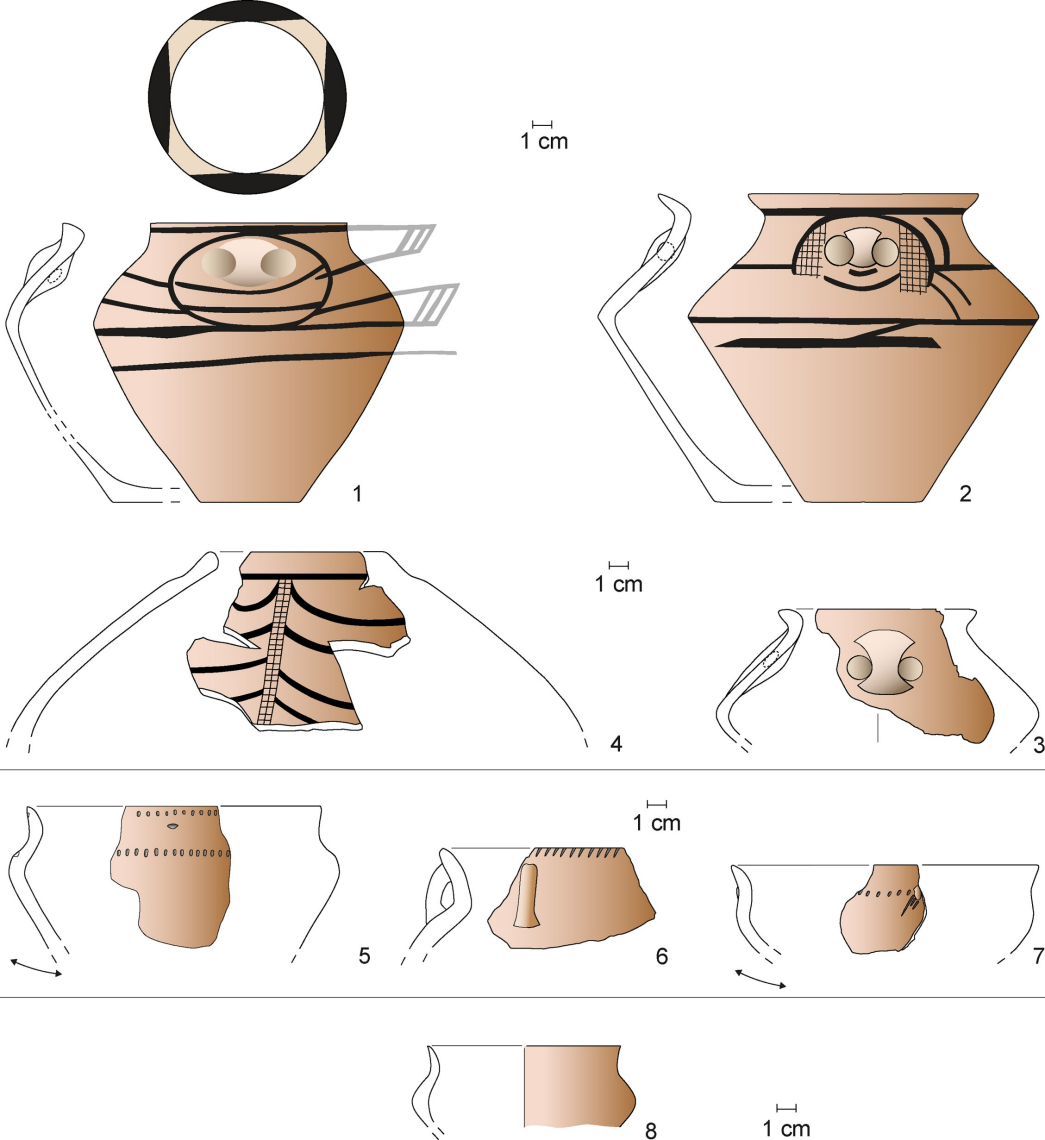
Maidanetske, ceramic inventory of mega-structure 3: Amphorae (1–3), pear-shaped vessel (4), pots made of kitchen ware (5–7), and pots made of table ware (8).

Pottery is distributed all over the mega-structure (Fig 11). For example, bowls, which are generally associated with consumption activities, are evenly distributed across the whole interior space of the mega-structure (Figs 11 and 12). Nevertheless, concentrations are visible in the northwestern and the southeastern parts. This might indicate different activity areas whose character might be detectable by functional differences of the involved morphological vessel types:

- Half-closed and closed vessels, which probably had storage functions, are concentrated in both already described zones (Figs 11–13). In the northwestern part of the mega-structure they are situated in the northeastern area east of the fire place. In the southeastern part they are concentrated in the southern corner beside the postulated entrance.
- Kitchen wares, which are associated with food processing activities, occur frequently in the southeastern part but have an additional distribution focus in the northwestern part of the building, mainly southwest of the fireplace (Figs 11 and 14).

In summary, the patterns of pottery distribution indicate food consumption in all parts of the mega-structure (bowls), food processing southwest of the fire place and along the southern walls of the southeastern part (kitchen ware), and food storage northeast of the fireplace and in the southern corner of the southeastern part. The lower fragmentation rate in the indicated zones supports our view that the named activities primarily took place in these parts of the mega-structure (Fig 11).

Remains of querns are again mainly concentrated in two zones of the mega-structure (Fig 11). Several fragments were found at the northwestern end of the building partly inside and partly outside of the external walls. Another concentration was observed in the central area of the southeastern part of the mega-structure, where the only complete quern was found.

In consequence, the different artefact distribution patterns seem to reflect the already discovered dual distribution pattern of the ceramics. We would particularly like to stress the contrast between the only partly preserved querns in the northwestern part and at least one complete and several fragmented querns in the central southeastern part. This might indicate that cereal processing only took place in the southeastern part of the mega-structure where also slightly more cereal remains were found (Fig 11). We interpret the fragmented querns as secondarily appropriated construction material, as also might hold true for a larger number of quarry stones, a boulder, and two unworked stone slabs (Fig 11). They are distributed in certain accumulations along the external walls and along the central axis of the mega-structure.

The spatial distribution of bones clearly reveals another focused activity area in the northwestern half of the mega-structure (Fig 11). In fact, the detailed bone distribution displays a half-circular density at some distance from the fireplace along the walls. This could indicate that the consumption of meat was restricted to the northwestern part of the mega-structure. Since heavily used areas within buildings are typically kept free from perishable waste, it is reasonable to assume that the bones within the building are from a meal just before the building was abandoned.

Other ground-stone artefacts include a polishing stone and a whetstone; both of which were found in the northwestern end of the building. With these artefacts, further activities are identified as taking place in the northwestern part, i.e. the polishing and the sharpening of tools (Fig 11). The distribution of the few flint artefacts (three pieces of debris and one flake) mirrors perhaps again the two larger activity zones in the northwest and southeast of the structure (Fig 11). This also holds true for remnants of textile production (Fig 11). In one concentration six fragments and one complete loom weight were found in the southern corner of the building. The second concentration consisting of a loom weight fragment and a spindle whorl was found in the western corner. The one fragment (foot and calf) of a large anthropomorphic figurine was deposited outside of the building along its northwestern narrow end and may indicate a certain kind of non-utilitarian practice linked to the northwestern part of the mega-structure.

In consequence, multiple domestic activities could be detected and localised. In the northwestern part of the mega-structure in addition to pyrotechnical activities at the fireplace, short-term storage, food preparation, meat consumption, textile production, and tool sharpening and polishing are identified. In the southeastern part of the mega-structure cereal processing, short-term storage, food preparation, and textile production took place. Food consumption is evident in both parts.

**Fig 15:**
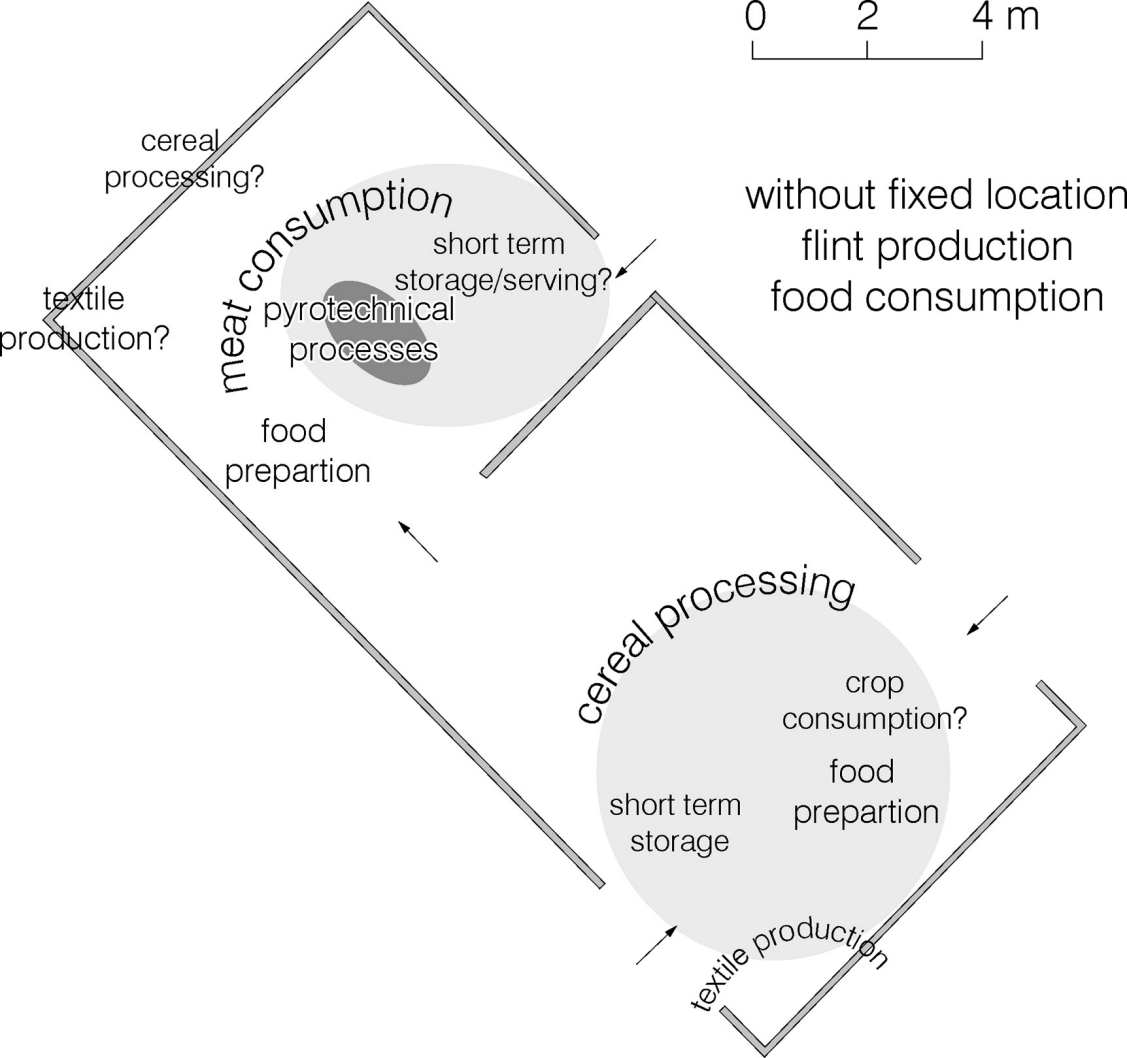
Maidanetske, reconstructed ground plan of mega-structure 3 with activity zones.

Comparing the architectural remains and the artefact distribution patterns, the ‘dichotomy’ between the northwestern and the southeastern part of the building is evident and crucial for the reconstruction of mega-structure 3 (Fig 15):

- The ca. 60 m^2^ of the northeastern part were constructed as a more or less closed space with walls up to 3.50 m in height and possible entrances from the outside, and a passageway to the southeastern part of the structure. The fireplace holds a central position within this roofed section. The main activities are linked to the consumption of cattle and pork meat, the sharpening of tools, and storage.
- The ca. 70 m^2^ of the southeastern part were constructed as an enclosed but unroofed space with lower walls up to 1.5 m in height in which cereal processing, but also food preparation, food consumption, short-term storage, and textile production took place.

In principle, our interpretation focuses on the difference between a roofed building in which meat consumption and pyrotechnic activities took place, and an appended unroofed enclosure in which activities including cereal processing were performed. Probably the spatial distribution of vessels (except bowls) with their concentration along the exterior walls indicates their original alignment. The difference between the roofed and the unroofed part of the mega-structure is reflected in the presence of charred *Stipa* awns in the southeastern part (Fig 11). Feather-grass is a plant of the steppe and might have entered the archaeological record due to its deliberate collection e.g. for matting [54] or attached to the fur of animals that visited spring-summer grasslands [55–57]. The presence of the tiny, charred, *Stipa* awns could be due to a taphonomical bias such as percolation from upper layers, but a direct radiocarbon date from another context in Maidanetske revealed to be contemporaneous to the site occupation (3969–3794 cal. BCE) [45].

In consequence, the differences in daub quantities between the northwestern and the southeastern part of the mega-structure have definitely architectural reasons and are not due to different degrees of burning. This interpretation is also supported by significant differences in activities between the two parts of the mega-structure. Whether this building was permanently inhabited or sporadically used will now be explored by comparison with other excavated dwellings.

#### Comparison with residential buildings

The architecture of mega-structure 3 (covering ca. 190 m^2^) differs substantially from that of dwellings 44 (77.5 m^2^) and 59 (42 m^2^). While the latter are characterized by massive platforms and indications of two storeys, the former is a one-storey construction. Consequently, much smaller amounts of daub were used for the construction of the mega-structure (house 44: 1–100 kg/m^2^ to mega-structure 3: <1–50 kg/m^2^) [46]. The remains of the internal architecture also differ. While in the mega-structure only a fireplace is documented, in each dwelling both a fireplace and an oven are present. Additionally, within the dwellings a podium and a bin were documented, which were missing in the mega-structure. While the division of the mega-structure into two parts could be seen as a reflection of the division of the main dwelling into two rooms, the aspect of roofing indicates clear differences: an open activity space which is much larger in size cannot be compared to a roofed and much smaller anteroom of a dwelling.

For the comparison of find inventories in Table 4 the objects are listed which were found within or in the direct vicinity of the excavated mega-structure 3. They are compared with the inventories of the two domestic houses 44 and 59. Additionally displayed are possible activities from which these finds might derive.

**Table 4:** Maidanetske, comparison of inventories of mega-structures 3 and dwellings 44 and 56.

object category	interpretation	house 44 (trench 51)	house 59 (trench 92)	mega-structure (trench 111)
flint artefacts	flint production	3 flakes	1 blade	3 debris 1 flake
anthropomorphic figurines (fragments)	ritual activities?	3	2	1
ceramic disk (fragment)	?			1
spindle whorl	textile production	1		1
loom weight (complete)				1
loom weight (fragment)				7
whet stone				1
Pounder		1	1	
rubbing stone				1
polishing/punching stone			1	
grinding stone: handstone	cereal processing?	2		
grinding stone: quern, lower		3		1
grinding stone fragments		6	5	9
quarry stone	construction?	1	4	21
stone slab	construction?		1	1
amount of pottery	food handling	61.4 kg	60.8 kg	37.9 kg
pottery density		0–29.5kg/m^2^	0–18 kg/m^3^	0–15.6 kg/ m^3^
engobe inside (open shapes)	food consumption	8%	11–16%	7–12%
portion of bowls*		24–35%	29–46%	17–26%
portion of cups*		16–32%	4–11%	4%
proportion of closed and half-closed vessels*	food storage?	34%	28–35%	56–74%
portion of kitchen vessels*		7–8%	3–8%	5–11%
proportion of kitchen ware kg / %	food preparation?	5.7 kg (9%)	1.4 kg (2%)	3.0 kg (8%)
n. of botanical samples		67	120	205 (214)
charred cereal grains (n. of finds)	food preparation?	14	24	20 (24)
charred cereal by-products (n. of finds)	food preparation?	3	11	2 (2)
charred potentially cultivated pulses (n. of finds)	food preparation?	32	0	0 (1)
charred *Stipa* awns (n. of finds)	matting? animal presence?	84	211	172 (200)

**Fig 16:**
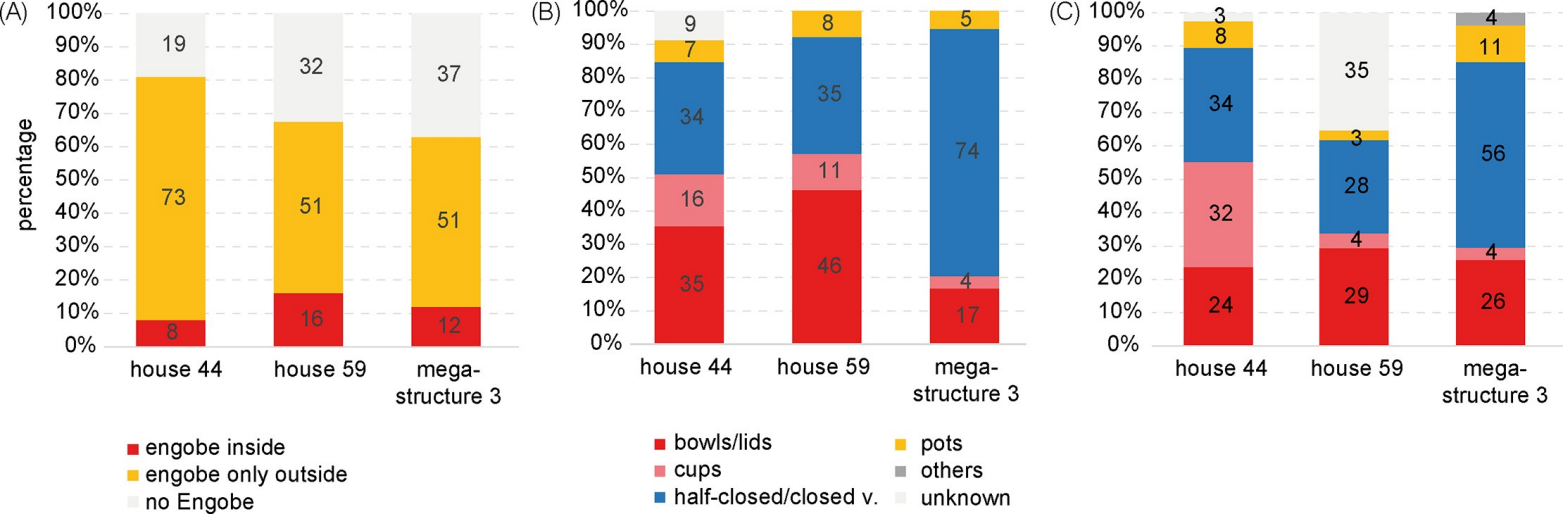
Maidanetske, comparison of pottery characteristics in dwellings 44 and 59 and mega-structure 3. (A) Location of engobe, (B) Frequency of morphological vessel classes according to the count of vessel units, (C) Frequency of vessel classes according to summed rim percentages.

Compared to the dwellings, mega-structure 3 contained overall a clearly lower amount (38 kg) and density (0.1–2 kg/m^3^) of pottery remains. The comparison of three buildings based on the percentage of engobe on the insides of sherds shows that bowl fragments are consistently ca. 8–12% of the ceramic assemblage (Fig 16). The counts of vessel categories for the dwellings show more or less similar percentages of vessel classes with relatively high amounts of both bowls/lids and cups (Fig 16). In contrast, in the mega-structure clearly higher percentages of half-closed and closed storage vessels were counted while cups for consumption are missing. A similar trend was detected also through summation of rim, belly and bottom percentages and the calculation of minimum individual vessel numbers (Fig 16). Accordingly, a minimum of 27 vessel units were associated with mega-structure 3 in contrast to 38 in house 44 and 68 in house 56.

Comparing the inventory of the mega-structure and residential houses, some possible differences concern, among other things, artefacts related to the textile production. While such finds in the mega-structure are represented by at least nine objects, they are very rare in both compared dwellings. In contrast, anthropomorphic figurines are rather less represented in the mega-structure than in residential buildings (the one piece that was found moreover was located outside the external walls).

Summing up, there are striking differences between dwellings and the investigated mega-structure concerning architectural design, internal organisation, and to a lesser extent also the kind and intensity of performed activities. Next, we will examine how representative the results achieved are by comparing mega-structure 3 with other mega-structures in Maidanetske and mega-structures from other settlements.

#### Comparison with mega-structures in magnetic plans

**Fig 17:**
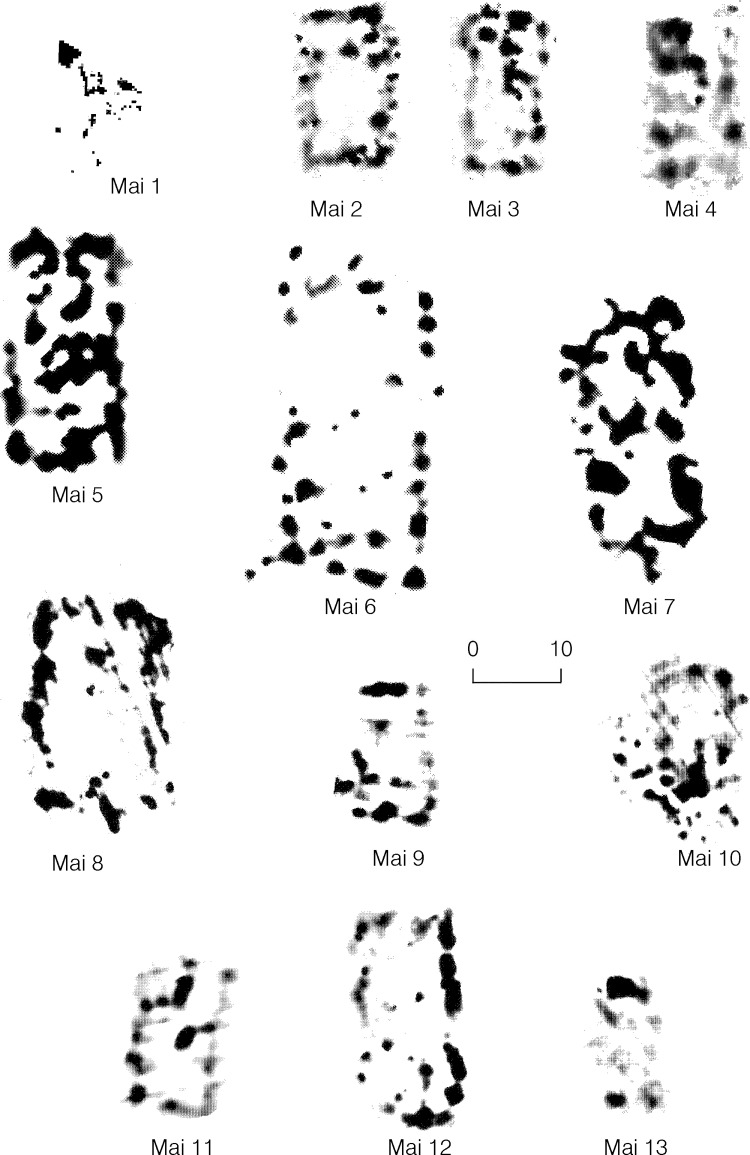
Maidanetske, magnetic anomalies of mega-structures 1–13.

In Maidanetske, 82% of the total settlement area has been surveyed by high resolution magnetometry, and thirteen mega-structures have been identified (Figs 1 and 17 and Table 2). Including mega-structure 3, seven of these buildings are located within the ring corridor of the settlement. In another five cases they were placed within radial trackways. Lastly, one construction was situated on a rectangular square in the east-northeast part of the settlement, of which, however, only a very small section could be recorded.

In addition to this positioning, the buildings show considerable size differences between 120 and 580 m^2^ and also a certain degree of architectural variability. Within the settlement of Maidanetske eleven of thirteen special buildings show an at least partially empty interior surface. Only in the case of mega-structure 5 is the laminar deposition of daub the case. In mega-structures 1, 3, 6, and 9 remains of internal partitions are visible. In eight cases point or point-like anomalies are visible along the central axis of the structure which most likely represent fireplaces. Thus, in Maidanetske, mega-structures show a considerable variability. Beside of partly roofed buildings like the investigated mega-structure 3 we may also need to consider that some of the mega-structures were completely unroofed and others completely roofed.

Also in magnetic plans of other Tripolye settlements, many mega-structures are characterised by low or no magnetisation of the interior space in contrast to the majority of residential buildings (Tables 1 and 2). Exterior, and in some cases also interior, walls are visible as linear (stripe-shaped) or spot-like magnetic anomalies. This characteristic is (at least partly) observed in 69 cases and raises again the question of completely unroofed mega-structures. In at least six cases, mega-structures with open interior space show internal subdivisions. Most frequent are structures with a smaller anteroom and a larger main room. A centrally located subdivision as reconstructed in the case of Maidanetske mega-structure 3 seems to be rare. According to the consistent evidence of the excavated cases from Dobrovody, Nebelivka, and Maidanetske, the frequently occurring dot-like anomalies along the longitudinal axis of the buildings usually represent repeatedly renewed fireplaces [30, 58, 59].

In contrast, thirty mega-structures show higher degrees of magnetization in large parts of their inner surface. Based on the measurements of the magnetic flux density and inversions of daub masses recorded during excavations it was possible to preliminarily estimate the masses of daub of four mega-structures from Maidanetske situated in the ring-corridor [46]. These estimations show for these special buildings clearly lower total masses and average masses per square-meter than for residential buildings. Accordingly, similar to the roofed parts of mega-structure 3, we can assume also in these cases single-level structures without the platforms characteristic for residential buildings.

Pits are associated with some mega-structures. In contrast to dwellings, these pits are in most cases not located on a gable side but on a longitudinal side of the building. In some cases, there is a row of single pits; in other cases longer pits stretch along the entire length of the building.

The comparison with a larger sample reveals a greater variability of mega-structures. At the same time, the comparison underpins the clear architectural differences between mega-structures and dwellings based on high versus low magnetization of the inner surface. Furthermore, where the mega-structures exhibit high internal magnetization there are still characteristic differences from residential buildings probably based on different masses of fired clay.

#### Comparison with other excavated mega-structures

Although the number of archaeologically investigated mega-structures is still low, the reasons for the above illustrated differences can be partly determined by examining those that have been excavated.

In the Precucuteni settlement Baia near Suceava in the Romanian Bucovina an ‘exceptional building’ was excavated which clearly differs from other dwellings of the site by its large size of at least 200 m^2^ and location in the centre of the settlement [38, 40]. According to preliminary reports the building was subdivided into eight rooms. The so far only very selective published inventory of this building consisted of grinding tools, chipped stone implements, and at least 200 vessels, among them a singular concentration of 25 vessels with anthropomorphic representations. Accordingly, as in Maidanetske mega-structure 3, many domestic activities are documented in Baia, although it is necessary to wait for a detailed publication of the find assemblage in order to make a more thorough comparison.

In the giant-settlement Dobrovody 144 m^2^ of a mega-structure were excavated [60]. This 46 x 26 m (ca. 1200 m^2^) large building structure is situated in an unbuilt ring-corridor of the site and appears in the magnetic plan as a rectangular structure with an enclosing wall and low magnetisation of the entire interior space [7]. During excavation debris of a lightweight built outside wall, remains of a poorly fired floor, and a round installation with three layers of repeated renewals were found. Only about sixty sherds and a fragment of an anthropomorphic figurine were documented. Based on these observations the excavators reconstruct an unroofed enclosure for unspecified ‘socio-economic purposes’. The case of Dobrovody shows that completely unroofed mega-structures likely existed in which obviously activities only with very low intensity took place.

**Fig 18:**
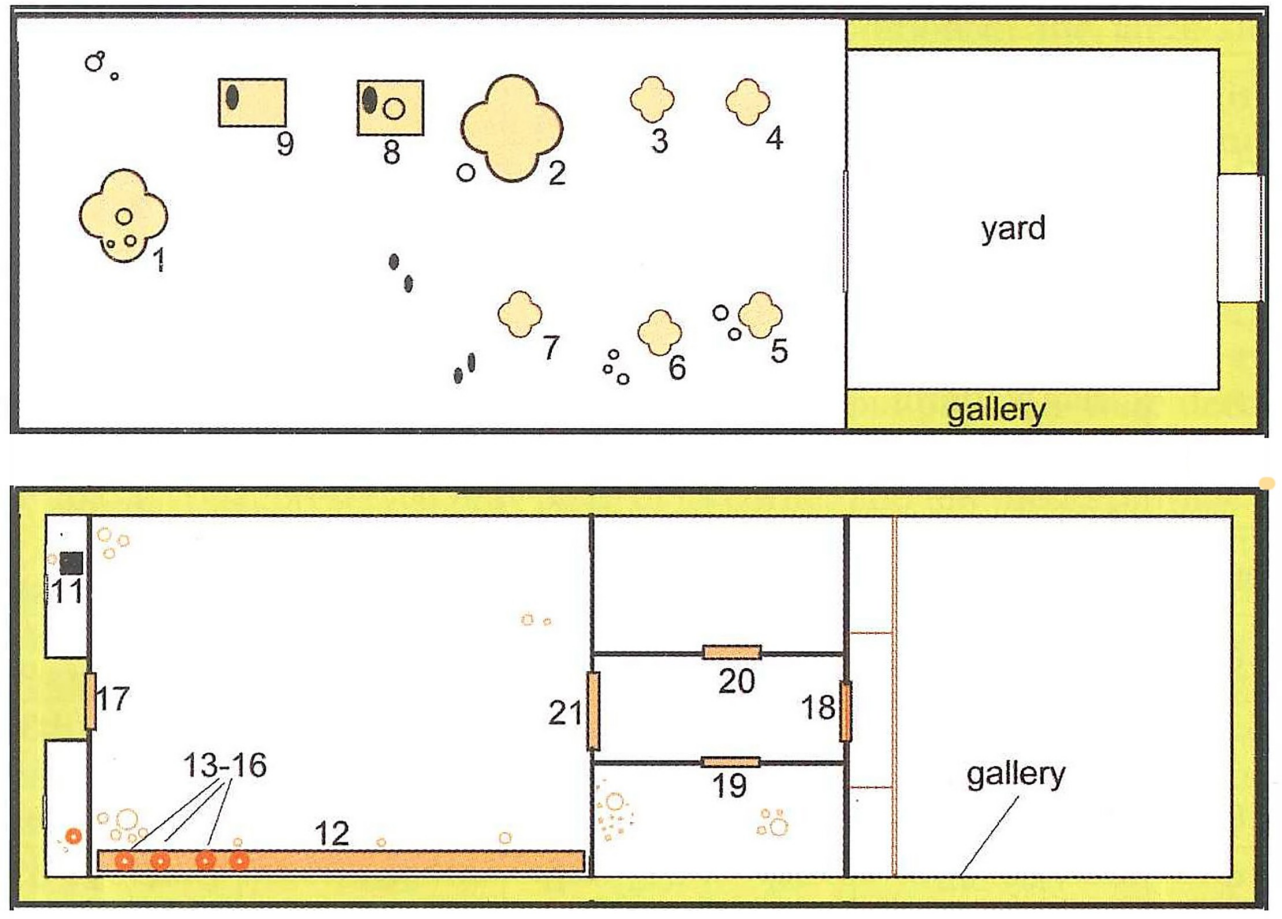
**Nebelivka, mega-structure, floor plans of the mega-structure (Reprinted from [59], Fig 1A under CC BY licence with permission from Editura “Constantin Matasă”, original copyright Natalia Burdo, 2014)** ground floor, b) presumed upper floor, 1–7 repeatedly renewed clay platforms (for pyrotechnical processes), 8–9 clay bins with grinding stones (grinding installations), 12 Podium, 13–16 large storage vessels (capacity around 50 l each), 17–21 thresholds of passages.

In the Tripolye B2 mega-site Nebelivka the so-called mega-structure with dimensions of ca. 1200 m^2^ (60 x 20 m) was excavated completely [9, 30, 31, 59]. This building was located in the eastern part of the settlement separated on a primary plaza in alignment with the inner row of houses of the ring corridor. It shows a division into two or three parts (Fig 18): The eastern probably unroofed part (20 x 20 m) consisted only of linear deposits of daub from an enclosing wall and was to a large extent free of finds. In contrast, considerably higher quantities of daub and other materials were deposited in the larger western part (38–40 x 20 m). It is not completely clear if this part was completely or only partly roofed. In addition, a possible multi-storey construction is under controversial discussion [30]. The interior design shows some elements of and a general arrangement similar to normal houses [53] like a “podium” along the southern longitudinal wall, clay bins, clay platforms, and internal walls. At least six cross-shaped and partly decorated fireplaces were arranged in two parallel rows in its middle part next to two grinding installations. The seventh, largest and also cross-shaped installation was located on the long axis of the building decentralized in front of the western gable wall [59].

The rather small find-inventory displayed no striking differences to residential houses apart from a small golden spiral, a few large storage vessels and several tokens. Some objects, such as anthropomorphic figurines were underrepresented in comparison to ‘normal’ buildings. According to Chapman, the associated find inventory is generally marked by a certain poverty [11]. Apart from a gold spiral, the inventory is not a material representation of any social hierarchy.

The case of Nebelivka confirms the finding that in mega-structures different 'domestic' activities took place. At the same time, the multiple occurrences of fireplaces and installations for milling of grain represent a remarkable difference from numerous other mega-structures.

#### Preliminary conclusions on the nature setup of mega-structures

Beside their high visibility in the public space of Tripolye settlements, different archaeological observations show clear differences between mega-structures and dwellings which support the idea of communal functions associated with mega-structures.

1. There are major architectural differences indicating that the daub deposits in the interior space of mega-structures rather represents remnants of wall debris and do not derive from raised platforms like in residential buildings.
2. Other differences in the architectural design are the presence of roofed and unroofed but enclosed spaces on a single level in contrast with the two-storey houses.
3. Completely unroofed mega-structures can be distinguished from partially to completely roofed mega-structures.
4. The spatial organisation of mega-structures clearly differs from that of domestic houses. Within normal dwellings activities took place in the enclosed private sphere, while the mega-structures had both an enclosed space and some kind of semi-public open part.
5. Within the three excavated Tripolye mega-structures fireplaces but no ovens were present.
6. While usually only one fireplace is present, in Nebelivka the occurrence of multiple fireplaces points to a special case.
7. Although numerous activities of ‘domestic character’ took place in the investigated mega-structure 3, the different character of the building is indicated by clearly lower densities of certain artefact classes. This concerns, for example, vessels for the handling of food and figurines for ritual activities.
8. Beyond these absolute quantitative differences, differences in relative abundances of vessels indicate a lesser importance of food preparation and consumption and a greater importance of short-term storage of food in the mega structures compared to domestic dwellings.
9. If there are pits associated with mega-structures they are situated not along the narrow but along the longitudinal side of the building.

According to the presented extensive evidences, mega-structures represent buildings whose architecture and locations in the settlement clearly differ from dwellings. The absence of ovens and the associated smaller find inventories suggest that mega-structures probably represent places which were not permanently inhabited.

### Categories of mega-structures

The following section uses a regional sample to systematically explore the extent to which different categories of mega-structures within Tripolye settlements can be identified that could correspond to different integrative social levels. This study will include the positioning of mega-structures within settlements, their dimensions, and architectural features. Based on these characteristics, a typological classification of Tripolye mega structures is proposed.

#### Identifying different positions of mega-structures in magnetic plans

In the site plans of Tripolye settlements, mega-structures are located not within concentric house rings, but within the ring corridor, radial trackways, or at the outskirt of the settlement. Some of them are located in empty spaces which are demarcated by lines of houses that we term ‘plazas’. These places are in a special location at the main entrance area of the settlements–singular primary plazas–and in other places, which are not so prominent for the whole site concept–secondary plazas.

**Fig 19:**
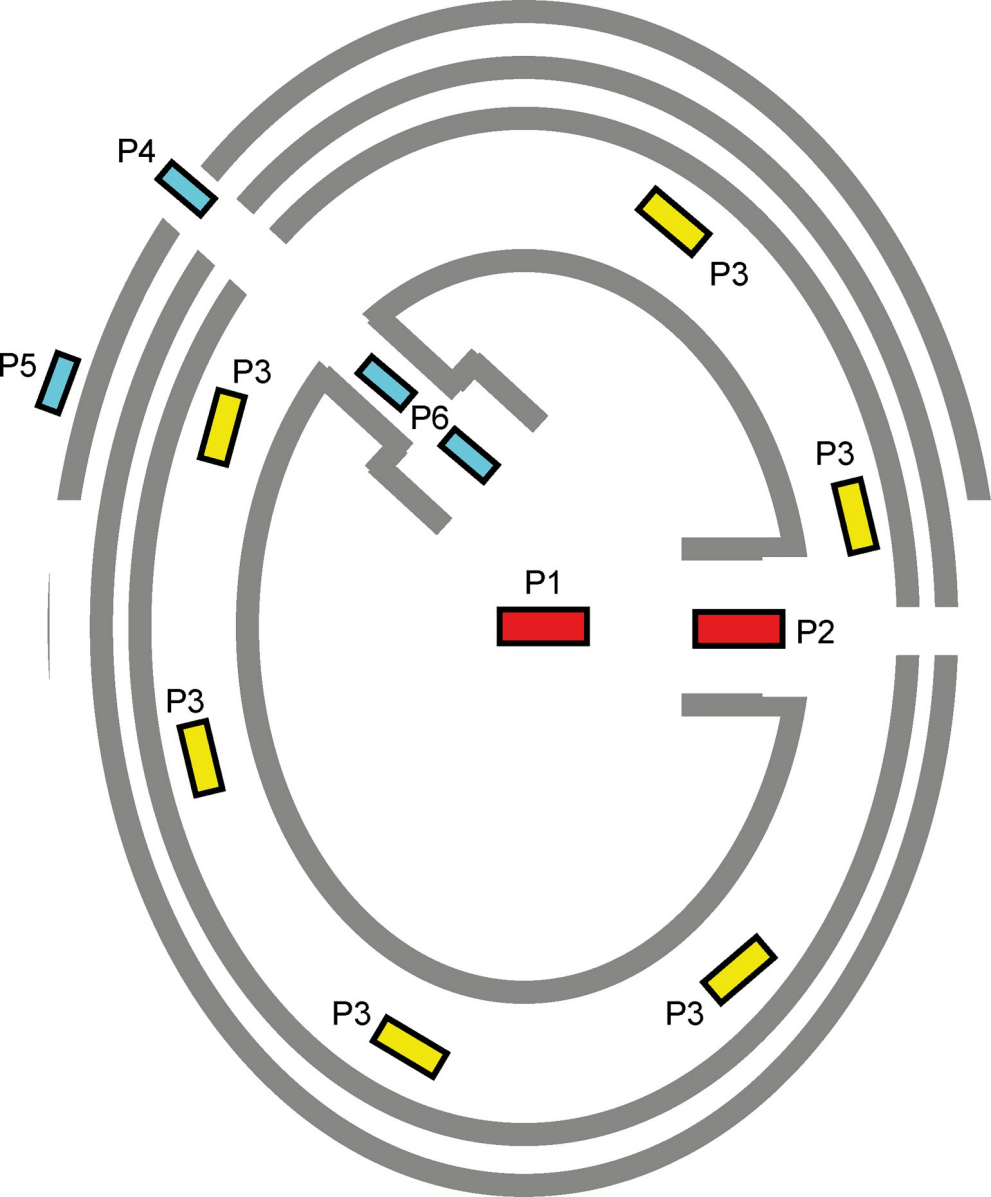
Schematic representation of the observed positioning of mega-structures in Tripolye settlements.

**Fig 20:**
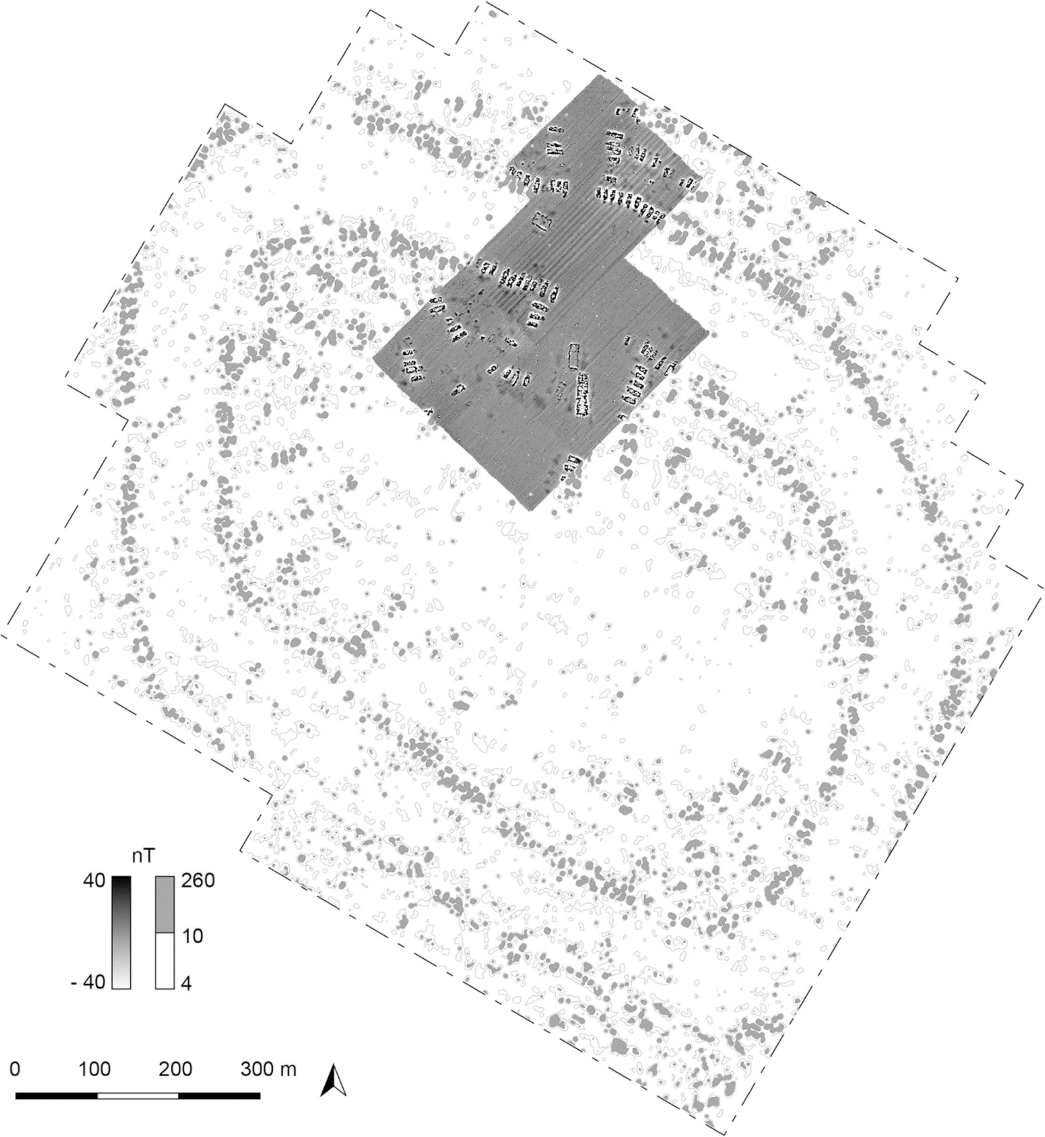
Hlybochok. Magnetic plan of the primary plaza in the north of the settlement (Reprinted from [34], Fig 152 under CC BY licence with permission from Sidetone Press, original copyright Renè Ohlrau, 2019).

The location of mega-structures within the site is one of the most important criteria for their identification in the magnetic maps. For classification purposes we consider six different categories of positons (Fig 19): **P1** in the centre of the settlement; **P2** on the primary plaza of the settlement (Fig 20); **P3** within the main ring corridor, mostly spaced at regular distances from each other; **P4** in the radial pathways; **P5** before the outer ring of the settlement; **P6** at the inner end of radial trackways, often in a kind of secondary plaza.

So far there is only one example for position P1 from the Precucuteni settlement Baia where, however, the integration of the mega-structure into the layout of the surrounding buildings is unclear [40]. In contrast, the mega-structures in the settlements Petreni and Trostiyanchyk represent rather a variant of the position P2 [43, 44]. In these cases, mega-structures were positioned at the end of the “plaza” and not in its centre.

In the analysed sample of settlements the number of different positions per site varies considerably depending on the scale of their investigation, size, regional setting, and dating (see below). In no settlement mega-structures are present at all positions. Rather, only single types occur or certain characteristic combinations, as exemplified by pairings of mega-structures in the ring corridor (P3) and at the outskirt of the settlement (P5) in Nebelivka and Volodymyrivka. In Maidanetske, contrastingly, mega-structures in the position P5 are missing while buildings in different positions on the radial streets (P4, P6) occur.

#### Measuring of mega-structures

**Fig 21:**
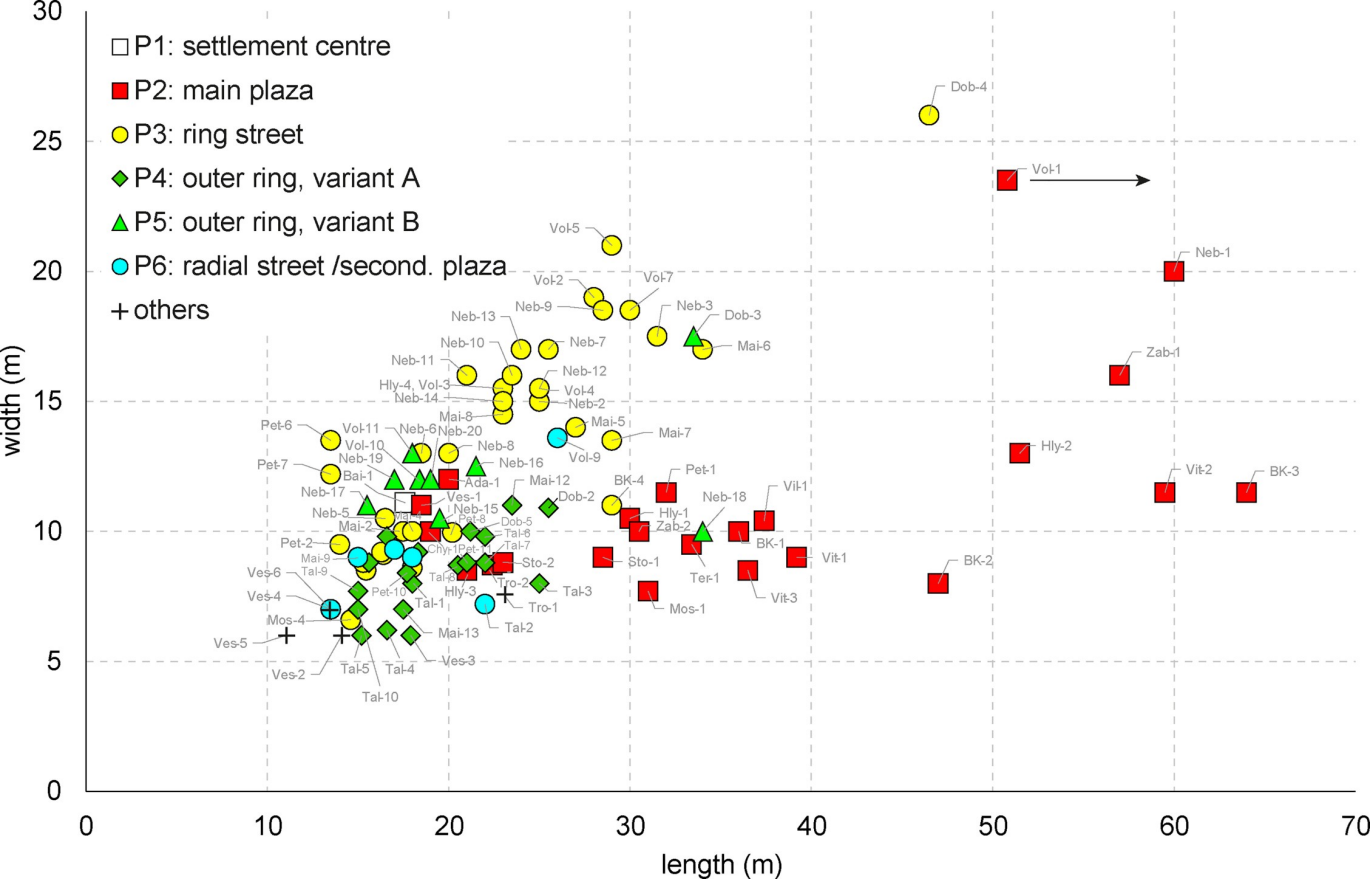
Scatterplot diagram of length and width of 104 special buildings from Cucuteni-Tripolye settlements between the Carpathians in the west and the Southern Bug-Dnieper Interfluve area in the east. **Mapped are the positions of the buildings within the settlements.** The buildings are coded with short names which are listed in Table 2.

In relation with the locations P1–P6, the mega-structures display differences in floor size; their size and proportions thus represent a second important criterion for a classification system. A scatterplot displays two sub-sets of buildings with different *dimensions* and *length/width ratios* (Fig 21). The first sub-set is exclusively located on primary plazas (P2); elongated length/width ratios between 1:2 and 1:4 and widths of not more than 12 m are characteristic, even if occasionally up to 16 m, and lengths between 18.5–65 m are observed.

In contrast, buildings of the second sub-set (P3–P6) are shorter and wider with length/width ratios lower than 1:2. Larger widths of frequently 20 and occasionally up to 26 m make the difference in terms of their size. Larger examples occur exclusively in the ring corridors (P3), smaller ones in or near radial trackways (P4–5) and outside the outer concentric house-ring (P6).

Mega-structures had large differences in floor area according to their position in the settlement. The biggest examples and at the same time the largest variance in floor area are found in constructions on primary plazas (P2), which range between 175 to 1200 m^2^ (mean 420 m^2^; median 315 m^2^, n = 24). Clearly larger than the majority of dwellings are also special constructions located in ring-streets whose floor sizes range between about 100 and 1200 m^2^ (mean 375; median 360 m^2^, n = 40). In contrast, clearly smaller are the vast majority of constructions situated in the outer ring of houses in the positions P4 and P5 and inside of the main ring in position P6: Their floor spaces range between 93–586 m^2^ (mean 200 m^2^; median 176 m^2^, n = 29).

Consequently, there are very clear differences in proportions and size of mega-structures between different positions in the settlements: The largest category is associated with primary plazas, a second category with the house-free ring-corridor, and a third category with other locations.

#### Typology and placement of mega-structures

**Fig 22:**
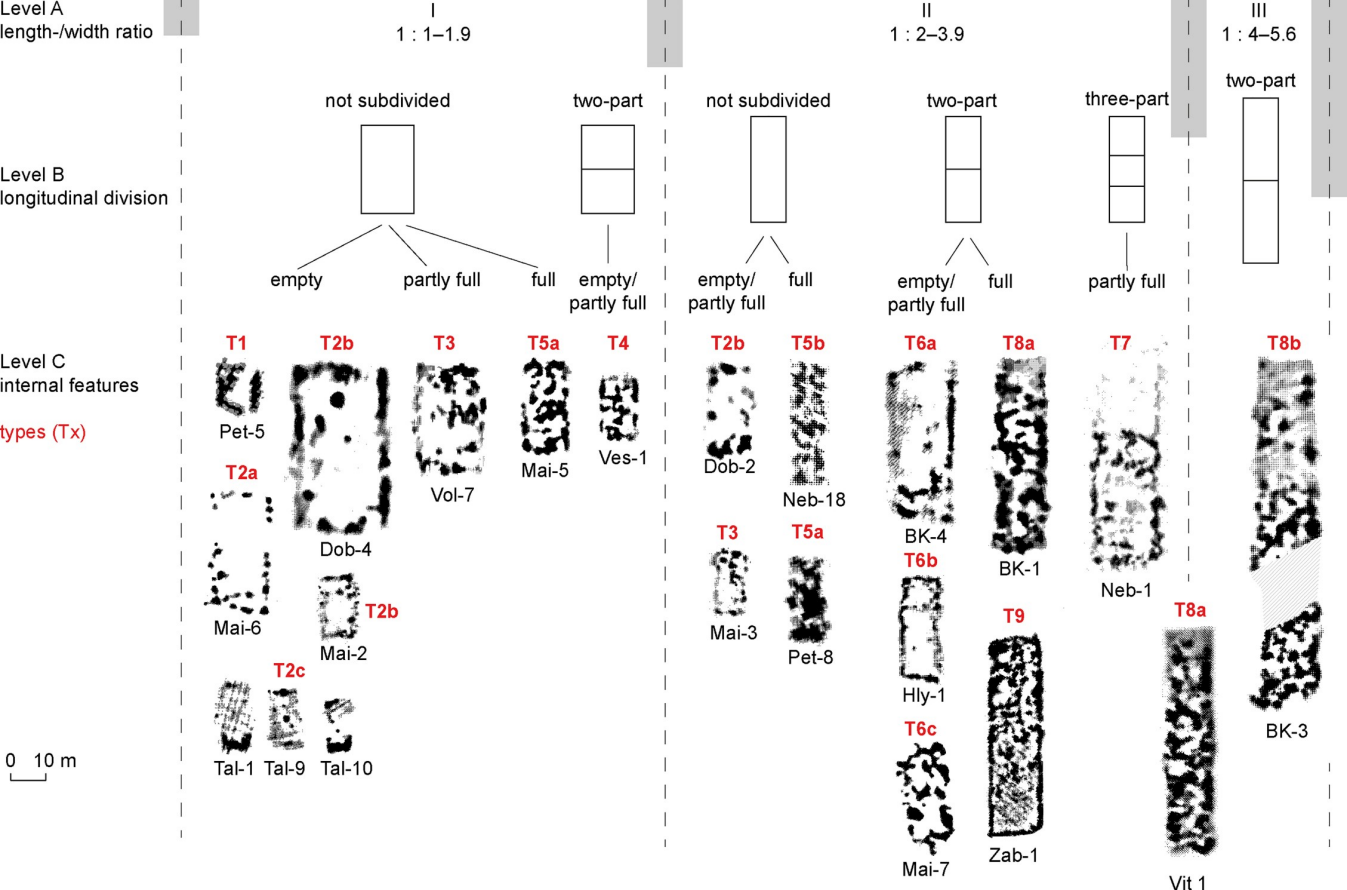
Sorting of Tripolye mega-structures by (A) length-/width-ratio, (B) division in longitudinal direction and (C) architectural characteristics and application of a type classification based on data of magnetic surveys.

To come up with the typology of mega-structures the *length-to-width ratio*, *internal partitioning*, and *other architectural features* were used as criteria. (S2 Table). As the length-to-width ratio could successfully be used to subdivide the mega-structures according not only to their size, but also to their location within the giant-settlements, we use this criterion as a primary tool for categorisation. Additionally, the main visible internal subdivision of the mega-structures is used as a secondary criterion. Differences in further elements are used as an additional tool for classification. Thus, a three-level typology allows a phenomenological division of the special buildings under discussion (Fig 22):

- Undivided building structures whose interior space is completely free of daub deposits or shows only some higher magnetized areas (types 1–3).
- Two- to three-part building structures whose interior spaces was also largely free of daub deposits (types 4, 6 and 7).
- One- to two-part building structures which show also in the interior space high magnetizations from daub deposits (subtypes 5, 8 and 9). Buildings of the latter group reach enormous lengths of up to 65 m.

**Fig 23:**
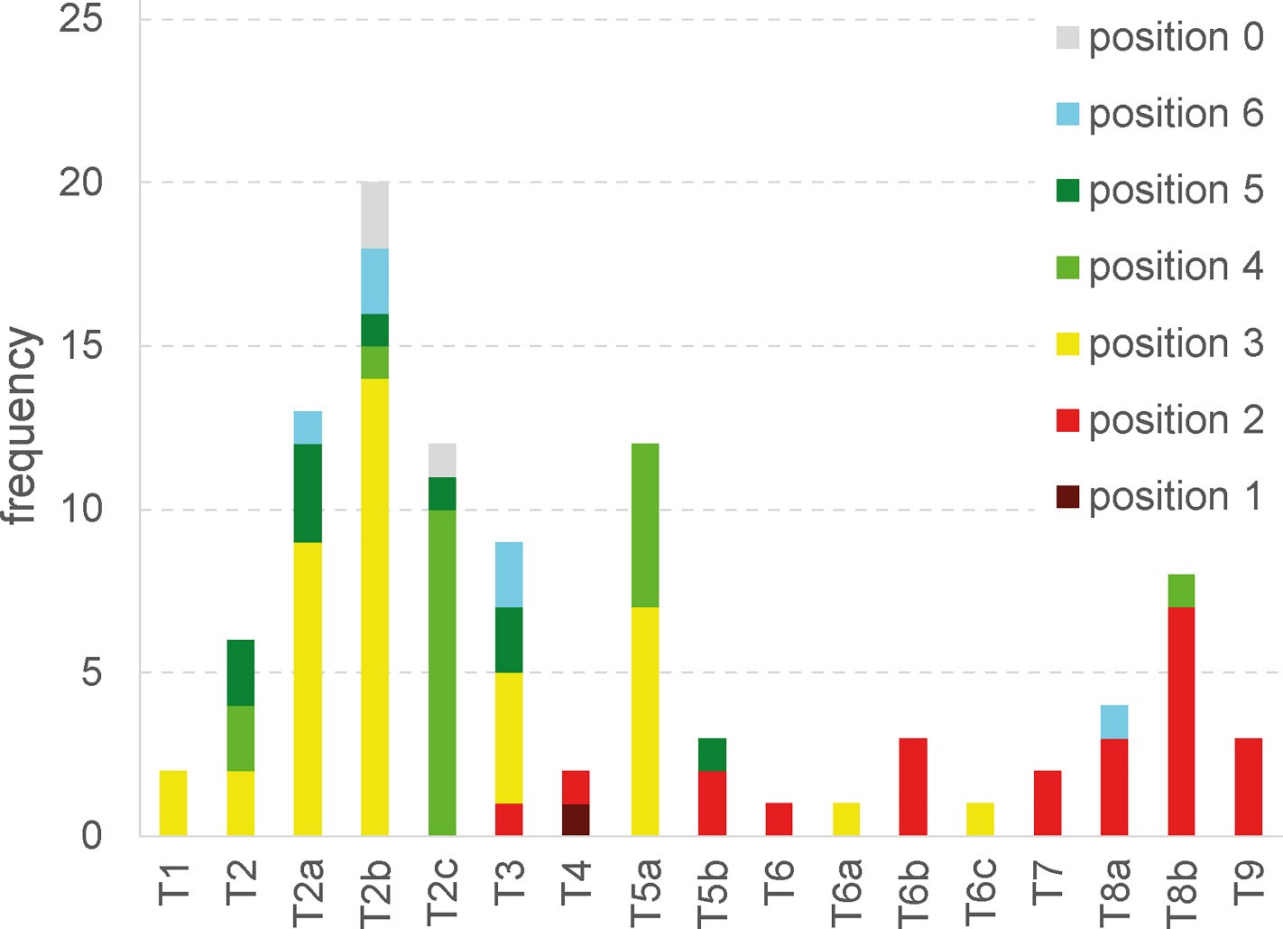
Bar chart displaying the frequency of positioning of the different types of special buildings in Tripolye settlements. The underlying data are available in Table 2.

With regard to the placement of the different types of mega-structures within settlements some clear preferences exist (Fig 23). Constructions with open interior surface (types 1, 2, 3 and 6a and 6c occur almost exclusively within ring streets (P3), at the outskirt of settlements (P4 and P5), and in radial pathways and secondary plazas (P6). In contrast, the open types (4, 6b and 7) and most of the overlong and interior magnetized representatives of types 5b, 8 and 9 are characteristic of primary plazas.

### Preliminary conclusions on categories of mega-structures

Based on the analysis of a large sample of mega-structures from Tripolye settlements with regard to their locations within settlements, their dimensions, and their architecture, the earlier made distinction [34] of two main categories of mega-structures can be confirmed and complemented by additional arguments. Accordingly, large and architecturally singular mega-structures on a plaza near the main entrance of the settlement can be distinguished from smaller repeatedly occurring mega-structures in other positions. The latter buildings in many cases had at least partly unroofed parts while the majority of the area of a larger number of the former group was roofed. Most likely this dichotomy corresponds to central institutions for the whole community on the one hand (first group), and institutions for parts of the community on the other hand (second group); this is also known from integrative architecture in ethnographically investigated non-ranked societies and referred to as ‘high-level’ (first group) versus ‘low-level’ (second group) architecture [27].

In addition to the distinction between low and high-level buildings, different categories within the low-level group have been identified based on their intra-site positioning and size. Accordingly, larger mega-structures in the ring-corridor can be distinguished from smaller buildings located at different positions on radial pathways and at the outskirts of settlements. However, in order to be able to discuss to what extent these different categories reflect also different integrative levels, use group size and diachronic developments need to be included in our analysis.

#### Use group size of mega-structures

The size of use groups associated with each low-level integrative building can be roughly estimated by using the numerical relation between the total number of dwellings and mega-structures within a settlement. The uniform distribution of low-level mega-structures within individual settlement is considered as key argument to assume their rough contemporaneity. For Maidanetske, on average, a 9 ha settlement area or 185 house-pit combinations were calculated as belonging to one mega-structure [8]. With an average house size of 72 m^2^ and a space requirement of 7 m^2^ per person this would correspond to a use group of 1900 persons. However, this should be understood as an absolute maximum value, since the contemporaneous occupation of all houses is a very improbable scenario.

For Maidanetske, a more realistic estimation of potential use group size is possible based on detailed stratigraphic analyses and Bayesian modelling of more than 80 ^14^C dates [25, 34, 45]. Accordingly, we need to take into consideration a settlement duration of 260–350 years with a population peak in the 38^th^ century BCE. Assuming an average duration of house occupation of 50 years, in settlement phase 3 the total number of contemporaneous houses amounts to 1550 houses. This would reduce the number of houses, which in purely arithmetical terms belonged to one mega-structure during Phase 3 to about 130 and the maximum use group size to around 1340 individuals.

**Fig 24:**
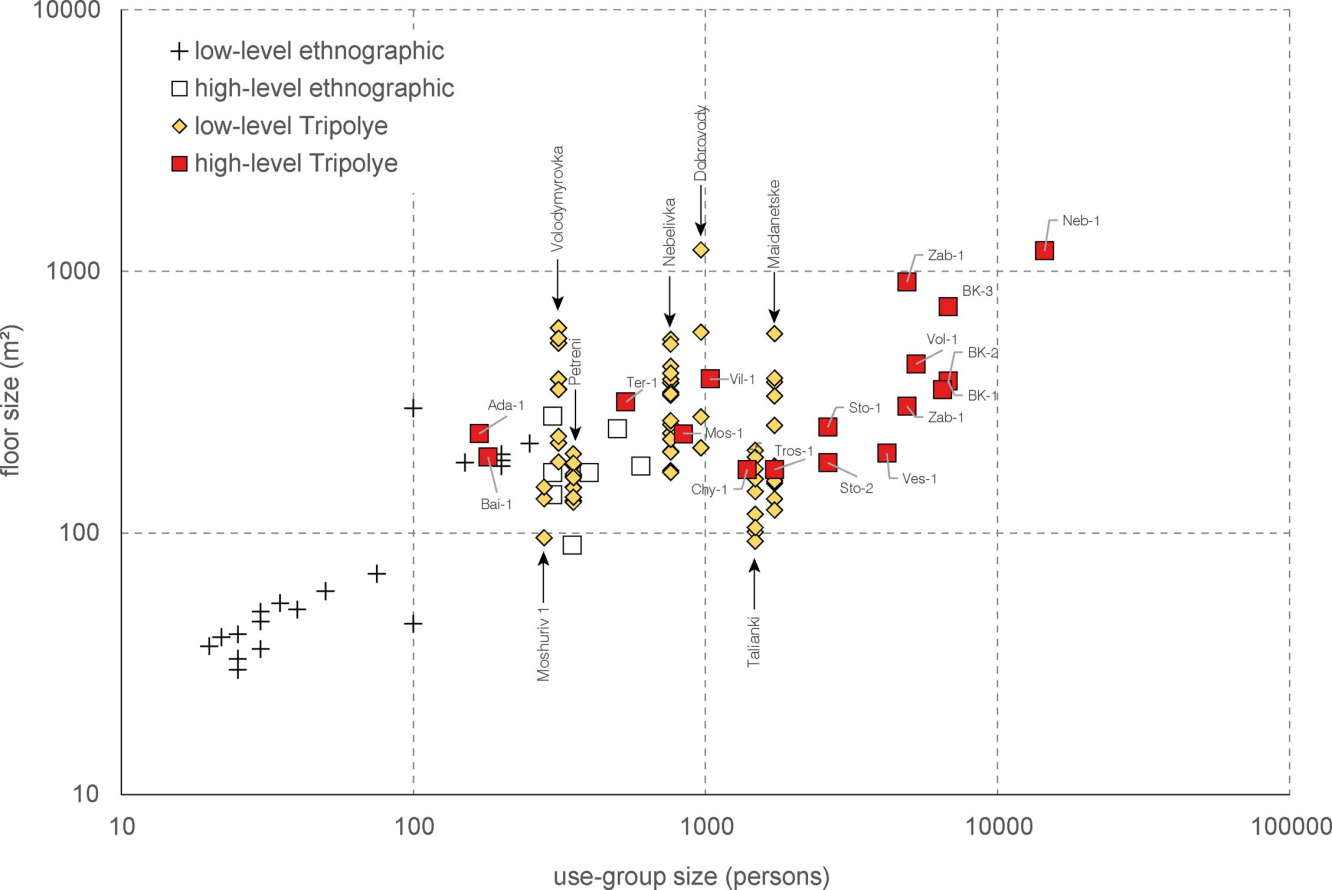
Scatter plot of floor area and estimated maximum use group size of 104 low-level and high-level special buildings in Tripolye settlements in comparison to the ethnographic sample after [26]. The underlying data for the estimation is displayed in Table 1.

Pronounced differences in the size of potential use groups of mega-structures are indicated by a high variability in the numerical relation between dwelling and mega-structures. The application of a similar calculation method resulted in significantly lower maximum use group size per special building between about 300 and 350 persons in Volodymyrivka and Petreni and 750 persons in Nebelivka (Table 1 and Fig 24). Similar values have also been calculated in the small settlement of Moshuriv 1 (280 persons) and the giant-settlement Dobrovody (600–750 persons). These are all significantly exceeded by the potential maximum use group sizes per mega-structure in Maidanetske (1340 persons) and Talianky (1500 persons).

To conclude, there is an enormous variability in the ratio of dwellings and mega-structures and the potential use group sizes of low-level mega-structures. This variability increases if high-level integrative facilities are also considered.

#### Mega-structures in time and space

In order to identify social processes in the archaeological record, it is necessary to analyse how mega-structures developed through time and how they vary across space. Thus, in the following section the attempt is made to explore basic trends in the development of mega-structures and to show aspects of their spatio-temporal variability.

#### The chronological development of mega-structures

Chronologically, a considerable variability in the frequency of mega-structures and their architecture is observed. The regular occurrence of mega-structures as a standard element of settlements begins at the latest in the period Tripolye B1–B2 between about 4200–4050 BCE. The highest frequency of special buildings was reached in the period Tripolye C1 between 3900–3600 BCE (Table 5). Considering additionally the different lengths of the periods, the highest density per 100 ha settlement area already existed earlier, in the period Tripolye B2 between 4050 and 3900 BCE.

**Table 5:** Frequency of special buildings in relation to the size of magnetically surveyed areas and to the duration of periods.

period (Tripolye …)	dating (BCE)	duration (a)	Measured area (ha)	number mega-structures	number houses/number mega-structures /100 ha	number houses/number mega-structures/100 ha/100 a
B1	4550–4200	350	20	1	5.00	1.4
B1-B2	4200–4000	200	111	11	9.91	5.0
B2	4000–3900	100	305	38	12.46	12.5
C1	3900–3700	200	242	37	15.29	7.6
C1 late	3700–3600	100	224	9	4.02	4.0

Until ca. 4200 BCE, mega-structures are very rare which might at least partly reflect the lack of magnetically surveyed sites belonging to these early Tripolye periods [61]. One of the earliest examples with a small-spatially structured ground plan was excavated in Baia south of Suceava in the Romanian part of Moldova.

Mega-structures are more frequent at the end of the period Tripolye B1 (Chyzhivka) and during period Tripolye B1–B2 in the last centuries of the 5th millennium. This development parallels the trend towards significant enlargement of settlements. In the Southern Bug-Dniester interfluve both constructions of the type 6b with partly open interior space (Zab-2) and oversized buildings of the types 5b, 8b and 9 with extensive daub deposits (Ter-1, Vil-1, Zab-1) occur. In Volodymyrivka, in the Southern Bug-Dnieper Interfluve, the so far earliest known ring-corridor buildings of the types 2b and 2c occur in addition to a large mega-structure with multiple rooms on the primary plaza of the settlement.

In the Southern Bug-Dnieper interfluve the development towards a rising number of special buildings arranged in the ring corridor and other positions of Tripolye settlements continued also in sites of the periods Tripolye B2 (Volodymyrivka, Nebelivka, Hlybochok) and Tripolye C1 (Moshuriv 1, Dobrovody, Maidanetske). In the Tripolye B2 period, we observe in the Southern Bug-Dnieper interfluve increasing architectural differentiation between buildings on the primary plaza and structures at other locations, even within small settlements. In earlier settlements like Volodymyrivka and Nebelivka buildings of the types 2, 6, and 7 with open interior spaces are the predominate types in all positions. Also on the main plaza in Hlybochok two buildings of the type 6b were situated. In addition, on this main plaza, an elongated oversized construction with highly magnetised interior space occurs for the first time in the region. Corresponding types of architecture might be adopted from the region west of the Southern Bug, where they occur already earlier in the period Tripolye B1-B2 (e.g. Zabolotne). The architectural diversity of the ensemble in Hlybochok might suggest that cases with multiple buildings on the same plaza represent different stages of settlement occupation.

**Fig 25:**
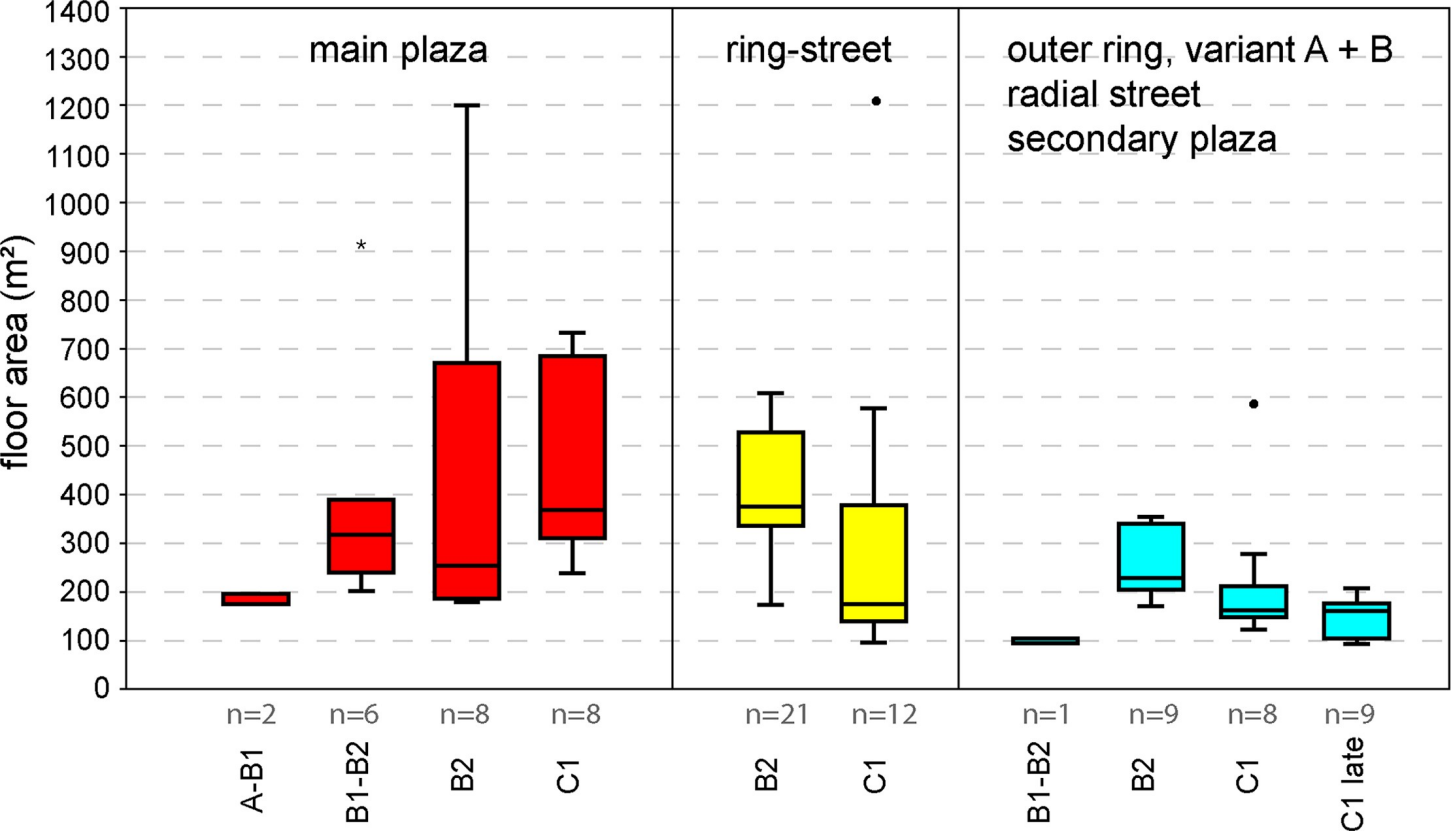
Boxplot diagram of floor areas of Tripolye mega-structures in a diachronic view differentiated according to positions within settlements. The underlying data are available in Table 2.

The observed architectural differentiation is accompanied by two reversal trends regarding the size of mega-structures in different settlement locations (Fig 25). Constructions in the positions P3–P6, which generally occur several times per settlement, became smaller between the phases Tripolye B2 (Volodymyrivka, Nebelivka) and late Tripolye C1 (Talianky). However, at least in the case of Talianky, this is due to the lack of special constructions in the ring corridor, which are usually larger than the here dominant buildings in the position P5.

In contrast, mega-structures on primary plazas (P2) show ever larger floor plans until the period C1 (Fig 25). However, as the regional comparison shows, this variability may also reflect regional differences due to imbalances of the sample. According to this, the average floor size of mega-structures on the main plaza is reduced from eastern to western Tripolye regions: from approximately 500 m^2^ (median 380 m^2^, n = 9) in region A to ca. 425 m^2^ (median 317 m^2^, n = 11) in region B, and ca. 219 m^2^ (median 220 m^2^, n = 4) in region C. However, the currently available sample from region C is still very small and represents only the periods Tripolye B1, B1-B2 and B2.

The described changes were accompanied by a clear trend towards rising number of contemporaneous houses and potential size of use groups which were associated with an individual mega-structure. While the maximum use group size of low-level buildings in the period Tripolye B2 is estimated in a range between 300 (Volodymyrivka, Petreni) and 750 (Nebelivka), there is a clear increase in the period Tripolye C1 with a total range between 280 (Moshuriv 1) to 1500 (Talianky) persons. This strong increase is even clearer in relation to the central, high-level mega-structures whose use groups potentially were the whole population of the settlement; in the case of Maidanetske this is estimated at between 10,000 and 20,000 people (Table 1).

The described trends are in particular remarkable if we consider the simultaneous reduction in size of special buildings. This applies specifically for buildings in the ring corridor (P3), radial streets (P4 and P6), and on the settlement outskirts (P5). In contrast, the size increase of the central mega-structures is rather plausible against the background of growing use group sizes.

Consequently, from middle to late Tripolye, important variations of the mega-structures developed. While originally only central, high-level mega-structures existed, around 4100 BCE additional low-level, ring-corridor buildings *appeared in the process of mega-site formation*. Subsequently, an increase in variation of both central and other mega-structures implies perhaps an increasing functional differentiation. In the later phase of the mega-site development, smaller mega-structures declined before they widely disappeared at the final stage. In the final stage the centralized power reflected in the primary plaza mega-structures not only survived, but extended its local dominance.

#### Regional differences

At the spatial macro scale, there are significant differences in settlement size and density within the Cucuteni-Tripolye complex. Accordingly, from west to east, the maximum size of settlements increases while the density of settlements decreases [13]. The majority of mega-sites are concentrated in the Southern Bug-Dnieper interfluve [32]. In the following section, the question will be examined to what extent these differences are also reflected in the use of mega-structures.

Pronounced regional differences exist with regard to the frequency of special buildings which only partially result from the unevenly distributed sample. With 74 (74%) cases, the majority of the examined buildings originate from the Bug-Dnieper interfluve (region A), while only 13 (12%) come from the Dniester-Southern Bug interfluve (region B) and 17 (16%) from the region between the Dniester and the foothills of the Carpathians (region C).

The reason for the different frequencies of mega-structures is primarily due to the absence of certain classes of buildings in the western part of the study area. In the regions C (Prut-Dniester interfluve) and B (Southern Bug-Dniester interfluve) mega-structures occur almost exclusively in the centres of settlements (P1) or on eccentrically located main plazas (P2). In contrast, the occurrence of buildings situated in the ring street (P3), in the outer house rings (P4 and P5), and in radial streets and secondary plazas (P6) is mainly limited to region A (Southern Bug-Dnieper interfluve).

One of the few known *exceptions* to this rule concerns the site Petreni in the north-western part of Moldova which was occupied ca. 3950–3700 BCE [62, 63]. In this site, special buildings are located at regular intervals in the ring road of the settlement similar to distributions recognized in the Bug-Dnieper interfluve area. However, the majority of these buildings show not the characteristic empty interior space of such ring structures, but have a completely magnetised floor plan. In regions B and C, types with low width-length ratios and open interior space are generally very rare. On the contrary, in these regions types with narrower proportions and magnetised inner surfaces prevail already in the period B1-B2. The same types were adopted later also in region A.

In principle, the regional differences display an increase in the presence and variability of mega-structures within sites from west to east. Surely this is linked to an increase in site sizes from west to east. Consequently, the diverging developmental trends of primary and secondary mega-structures reflect the necessity to negotiate old and new institutional governance of giant-settlements.

## Discussion: Integrative architecture

### What are mega-structures?

Until now, the main argument for the public character and communal functions of mega-structures consisted of the placement of the buildings in highly visible positions within Tripolye settlements. This was demonstrated for the site of Maidanetske by René Ohlrau based on view-shed analysis [8]. This high-visibility aspect applies not only for buildings placed in the ring corridors, but also for those in radial pathways. Recently, this aspect can also be realised in the most impressive ensemble of three huge mega-structures from Bilyi Kamin which were positioned next to each other in landscape-dominating position on a widely visible promontory showing definitely some kind of monumental character [41].

Newly identified aspects of mega-structures are their major differences compared to normal houses. These differences concern architectural design such as the presence of roofed and unroofed parts and the terrain level construction. Other differences concern their semi-public character and different spatial organisation. Rather unusual aspects are also represented by at least ten tokens, several pendants, and a small golden spiral. Additionally, comparatively low artefact densities and the absence of ovens suggest that mega-structures were not or not permanently inhabited.

Besides these striking differences, there are also important common aspects with normal houses. This applies, for example, to partly decorated and repeatedly renewed fireplaces which obviously represent an important standard element not only of houses but also of mega-structures. These installations are interpreted as communicative cores of Tripolye houses due to their prominent placement on the central axis of the buildings [37].

Shared aspects of dwellings and mega-structures also concern numerous ‘domestic’ activities which were identified in both types of buildings such as storage, preparation and consumption of food, the milling of grain, the craft production and specific ritual activities, represented by vessel assemblages, animal bones, botanical macro-remains, querns, artefacts for textile production, and anthropomorphic figurines.

Important for our interpretation of mega-structures is comparison with integrative buildings in 28 ethnographically documented societies from North America, South America, New Guinea/Oceania, and Africa. In ethnographic situations, a poly-functional character and a frequent use for both ritual and non-ritual activities have consistently been observed [26, 27]. This use can include various aspects such as information sharing, joint decision-making, administrative purposes, body cleansing, stockpiling, or the redistribution of goods. Consequently, performing day-to-day ‘domestic’ activities in integrative facilities is the normal state rather than the exception.

Thus, we do not consider the various domestic activities which have been proven for the excavated examples from Maidanetske and Nebelivka as a reason to question the expected public functions. In contrast, in our opinion, the described wide range of activities associated with Tripolye mega-structures completely prevents the interpretation of these constructions as specialized production or central storage facilities, but rather indicates their communal nature as a place for integrative action. Such integrative actions could include feasting during which certain rituals of consumption were performed to share surplus, to acquire prestige and social power, or to maintain existing inequalities [64]. In another context in Maidanetske feasting activities have been already proven connected with the deposition of two cattle skulls and numerous bowls at the bottom of a pit [37]. Generally, in Tripolye mega-sites we can assume an increased importance of ritual and ceremonial activities that provide a frequently observed mechanism for reducing scalar stress in large human groups [15]. This is also not contradicted by the rarity of anthropomorphic and zoomorphic figurines in the mega-structure, since these objects might mainly be related to specific ritual activities linked to the domestic sphere of normal households.

### Mega-structures and social organization in Tripolye settlements

As shown above we identified four different locations of mega-structures within the evaluated sites, which probably can be placed in a kind of hierarchical order:

1. Mega-structures at the main entrances of giant-settlements are usually the largest and integrate functions for the organisation of the whole mega-site community.
2. Mega-structures in the ring-corridors are usually smaller and integrate organization efforts of only segmented parts of the mega-site community.
3. Mega-structures in the radial trackways are also smaller and they integrate purposes which might be associated with the control of the trackways or of subsequently added quarters.
4. Mega-structures at the periphery near the outer house-ring are of similar size to those mentioned in no. 3.

In our view, these four different spatial locations reflect some kind of system of hierarchical levels of social and political organisation related to different use groups. Therefore, buildings on these different levels show also different architectural characteristics and dimensions. It is not surprising that with the increasing size of Tripolye sites, the number of organizational levels also increased. As we already stated, high-level mega-structures associated with the whole settlement were complemented by low-level buildings for smaller sub-groups successively during phases of settlement growth. The new integrative architecture is a reflection of the situation in giant villages where it is impossible for everybody to know everybody and therefore the means of political organization have to increase.

Potentially, the different categories of mega-structures were related to different integrative activities or different kinds of local sub-groups which constituted as a whole a settlement community. This hypothesis is supported by the striking pairings of buildings in the ring-corridor on the one hand and on the outskirts of the settlement on the other hand observed in Nebelivka and Volodymyrivka. Also the pattern in Talianky points in the same direction where buildings in the ring-corridor are completely missing and only small buildings in radial trackways occur.

With regard to the question who actually took part in the integrative actions within mega-structures, we observing a systematic discrepancy between the estimated use group sizes of Tripolye mega-structures and use groups of integrative buildings which were documented in the cross-cultural ethnographic sample (Fig 24). Use group sizes in ethnographic case studies are usually much smaller and never exceed maximum 600 people even in high-level integrative units [26, 27]. Thus, in particular in the case of Tripolye giant-settlements with their large estimated use group sizes, we need to take into account that only a limited part of the total population was involved in the decision-making processes in mega-structures or that preceding estimations of numbers of inhabitants resulted in to high numbers.

Indirectly this supports the ideas that also at lower levels than larger districts or quarters additional integrative institutions existed which could be, for example, related to house clusters [10]. Additionally, mega-structures in less prominent positions such as those buildings at the fringe of the settlement area could represent lower levels of decision-making or different sub-groups.

### Mega-site collapse and the loss of middle-lower decision-making levels?

If we accept the arguments regarding the integrative nature of mega-structures and the different hierarchical scales represented in the different positions of mega-structures in Tripolye settlements, we are able to deduce also the societal institutions behind these structure and to track their development. Accordingly, integrative architecture existed already since the early stages of Tripolye societies in the first half of the fifth millennium but were for a long time exclusively related (or visible) to the organisational level of whole communities much smaller in terms of population size [40]. Related to the beginning of the formation phase of giant-settlements in the last centuries of the fifth millennium BCE, villages of partly already enormous size emerged which regulated decision-making further on the level of the whole community.

**Fig 26:**
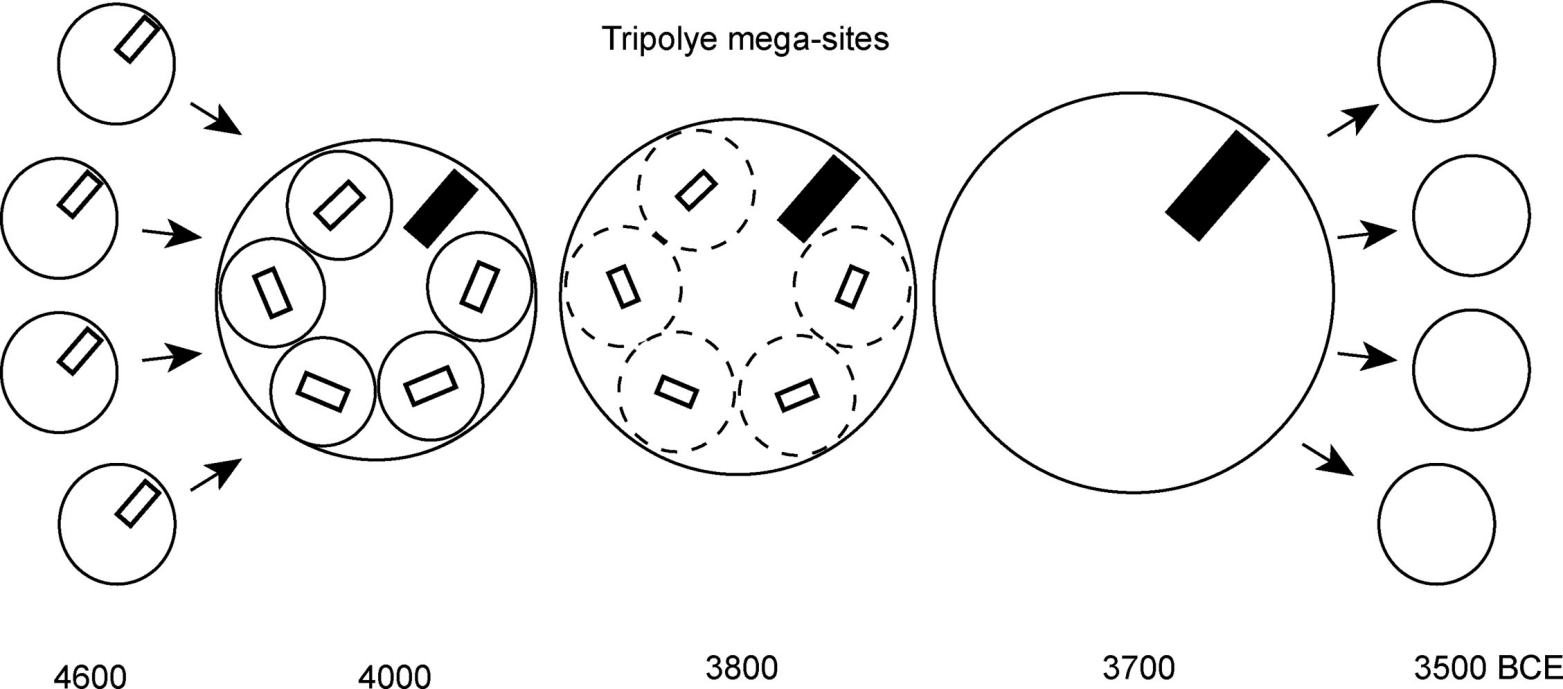
Schematic reconstruction of transformation processes of political integrative institutions and architecture in Tripolye settlements. Around 4600 BCE–In small settlements, integrative activities took place in central mega-structures. Around 4100/4000 BCE–Giant-settlements developed through the fusion of smaller communities, which maintained their integrative institutions and established high-level central institutions for the entire settlement. Around 3900/3800 BCE–Ongoing centralisation of integrative activities is indicated by increasing functional and architectural differentiation between high-level and middle-low-level integrative buildings. Around 3700 BCE–Increasing centralisation of social and political activities led to the loss of the middle-lower integrative decision-making levels. Around 3650/3500 BCE–Disintegration of Tripolye giant-settlements and re-emergence of a dispersed settlement system without (visible) integrative architecture.

The emergence of additional integrative social and political institutions below the level of the entire Tripolye giant-settlement is most likely the result of the fusion of different local units which attempted to maintain their local organisational structure (Fig 26). Within the Tripolye giant-settlements these units might be equated with some kind of ‘quarters’ or ‘districts’ [65]. Thus, the uniform distribution of mega-structures within Tripolye giant-settlements probably reflects the planned and equal process of the fusion of different smaller communities in giant-settlements. The lower decision-making levels are as necessary to keep the mega-site manageable as the central level for the whole mega-site.

Therefore, upon their arrival in the new settlements, representatives of the different segments or sub-communities of mega-site populations founded new central institutions together, which are represented in the mega-structures on the main public plaza of large settlements. In these high-level structures, the concerns of the whole community were managed; these central institutions were necessary in order to manage information flow and decision-making and to reduce scalar stress at the organisational level of the whole settlement [66, 67]. In terms of population size these new institutions are related to significantly larger populations quantified, for example, in the case of Maidanetske to more than 10,000 inhabitants [25].

In the following 400 years, until about 3600 BCE, the political integrative institutions underwent substantial transformations. As is suggested by reverses in the size and architectural development of mega-structures in different positions, integration at the level of sub-communities lost importance while high-level integrative institutions at the community level became more important. As a result of this process, probably more political decisions were made at the community level while the sub-communities became focused on other integrative activities. Consequently, the institutional differentiation was accompanied by increasing architectural and functional differentiation. The differentiation process described was probably the result of increasing integration at the community level and a blurring of social boundaries between the original settlement units. Thus, we see a centralization process in which obviously decreasing numbers of people were involved in political decisions.

Although in Talianky the detection of central mega-structures is pending due to inaccessibility, the absence of mega-structures in the ring-corridor seems to indicate that low-level integrative institutions had largely lost their importance at the end of the sequence of Tripolye giant-settlements. Obviously, the integrative action and power, which was in earlier sites much more equally distributed within a segmented society, passed over to central institutions.

The observed centralization certainly concerned not only political decision-making processes, but probably also the ritual dimension of mega-structures and the composition of the group of people involved in shared consumption of surplus. Indeed, the ritual importance of mega-structures is reflected only to a lesser extent in the composition of the artefact inventories. However, increased ritual importance is indicated inter alia by an increasingly elaborated architectural design of the main plaza in the ground plan of settlements as clearly demarked public square spatially separated from the ring corridor. Overall, we regard the food, which was consumed in mega-structures, as surplus that has been removed from the domestic context and consumed in commensal meals by a now much smaller group of people. This advanced centralization of political decision-making, ritual activities, and possibly also the redistribution of a low-scale surplus might indicate the emergence of certain hierarchies or a change in cooperative modes of production and consumption. The observed regional differences are probably partly the result of different formation processes of giant-settlements, which seem to correlate with a general trend towards smaller settlements west of the Southern Bug. Accordingly, large settlements to the west of the Southern Bug are perhaps to a lesser extent the result of inter-site-mobility but rather of natural population growth? Additionally, there is certainly an overall lesser degree of population agglomeration and a certain temporal focus of the examined sample on periods in which there were no mega-structures in secondary positions to the east of the Southern Bug.

Consequently, the developing centralisation in decision-making might reflect an increase in social inequality and resulting imbalances of group interests within large Tripolye communities should be taken into account as a critical factor for the disintegration of Tripolye giant-settlements. This is especially the case since previously discussed factors such as the non-availability of firewood and timber [45], insufficient carrying capacity [68] and soil depletion [69] can meanwhile be excluded with certain probability. Instead, scalar stress resulting from dysfunctional collective decision-making and the non-acceptance of emerging hierarchies could be important determinants for the end of the mega-structures beside of other possible factors such as climatic change [70] and epidemics [71].

From the point of view of the population sizes of ca. 10.000 +- 5000 inhabitants reconstructed for Tripolye mega-settlements [8, 14, 25], a high degree of scalar stress must inevitably be presupposed, which had to be reduced by effective mechanisms. The mega-structures discussed in this paper represent the aspect of sequential decision-making as one of the possible mechanisms for managing social complexity and reducing scalar stress [15]. According to our data, this bottom up mechanism increasingly turned into an increasing dominance of centralized decision making in contrast to institutionalized decision making on different scales.

The increased social complexity as prerequisite for the described centralization process is only weakly supported through the archaeological data or masked by selective preservation conditions. Anyway, in Tripolye mega-sites, for example, significant differences in floor size of houses existed beyond apparently uniform find-inventories [8]. A certain complexity is also indicated by increased specializations in pottery production [72], textile manufacture [10], supply with stone raw materials [73], and possibly food production [10]

Decisive for the failure of the just emerged hierarchical decision making, was in our view the continued predominant ideology of social autonomy of segmented lineages. Thus, in Tripolye mega-sites we don’t see the generalized failure of the dispersed (bottom up) decision making structure but rather the result of social contradictions arising from dissatisfaction and non-acceptance with central (top-down) decisions of centralized institutions of the settlement. Conceivable areas of conflict might include access to arable land nearby the settlements or social asymmetries resulting from the growing social complexity and specialization. The value of mega-structures for the reconstruction of social space within Tripolye settlements lies in the fact that this special category of buildings reflect in a very specific way different aspects of the social organisation in segmentary Tripolye societies: In Tripolye giant-settlements they reflect different stages of social integration of societal sub-groups and signify social tensions and organizational insecurities within the concept of mega-sites. Additionally, they can be used to reconstruct central institutions within these villages. Consequently, the configuration of integrative architecture can serve as a proxy for different historical states and processes including political decision-making structures, ritual integration, surplus redistribution, and different scales of political and economic organization.

## Supporting information

S1 Table Maidanetske, botanical macro-remains from trench 111 and mega-structure 3. (DOCX)

S2 Table Type description of the typology of Tripolye mega-structures. (DOCX)

S1 File Maidanetske, trench 111, OxCal codes and results of Bayesian modelling. (DOCX)
