# Supplementary material for: Governing Tripolye: Integrative architecture in Tripolye settlements
Source: PLoS One. 2019 Sep 25;14(9):e0222243. doi: 10.1371/journal.pone.0222243 (PMC6760824; doi:10.1371/journal.pone.0222243)
Supplement: S1 Table — (DOCX) [file pone.0222243.s001.docx]

# **S 1 Table. Maidanetske, Botanical macro-remains from trench 111 and mega-structure 3**

|  | **Trench 111** |  | **Only mega-structure 3** |
| --- | --- | --- | --- |
| **volume of soil** | 2395 |  | 2025 |
| **n of features** | 22 |  | 14 |
| **n of samples** | 214 |  | 205 |
| **Cereal grains** |  |  |  |
| *Hordeum* sp., grain | 1 |  | 1 |
| *Triticum* sp., grain | 3 |  | 3 |
| Cerealia indet., grain | 20 |  | 16 |
| **Cereal by-products** |  |  |  |
| Cerealia indet., spikelet | 2 |  | 2 |
| **Pulses** |  |  |  |
| Fabaceae (cult.) | 1 |  | 0 |
| **Gathered plants** |  |  |  |
| *Stipa* sp., awns | 200 |  | 172 |
| **Ruderal and segetal vegetation** |  |  |  |
| *Chenopodium album* | 60 |  | 59 |
| *Chenoodium polyspermum* | 1 |  | 1 |
| Chenopodiaceae | 13 |  | 13 |
| cf. *Avena* sp. | 1 |  |  |
| cf. Panicoideae | 1 |  | 1 |
| cf. Poaceae | 1 |  | 1 |
| *Vicia* sp. | 1 |  | 1 |
| **total finds** | 305 |  | 270 |
| Indeterminata | 109 |  | 74 |
