# Supplementary material for: Governing Tripolye: Integrative architecture in Tripolye settlements
Source: PLoS One. 2019 Sep 25;14(9):e0222243. doi: 10.1371/journal.pone.0222243 (PMC6760824; doi:10.1371/journal.pone.0222243)
Supplement: S2 Table — (DOCX) [file pone.0222243.s002.docx]

# **S2 Table. Type description of the typology of Tripolye mega-structures.**

| Type | length-/width ratio | division in longitudinal dir. | architecture |
| --- | --- | --- | --- |
| 1 | c. 1: 1 | - | walls are displayed as highly magnetised stripes or dots while the interior space is free of daub |
| 2a | I or II | - | walls: highly magnetised stripes or dots; interior space: free of daub |
| 2b | I or II | - | like 2a + round anomaly (installation) located decentralised in the longitudinal axis of the building |
| 2c | I or II | - | like 2a or 2b but within the embracement high magnetisation only at the narrow sides |
| 3 | I or II | - | like 2a + burnt wall debris concentrated in one quarter of the interior space |
| 4 | I | two-part | like 2a, central division in two equal parts and perhaps further division |
| 5a | I or II | - | burnt wall debris on the entire surface |
| 5b | II | - | like 5a but longer |
| 6a | II | two-part | like 2a with small anteroom |
| 6b | II | two-part | like 2a with larger anteroom (in position 2 oriented towards the settlement entrance) |
| 6c | II | two-part | like 2a with central division in two equal parts |
| 7 | II | three-part | on the narrow side open empty part towards the settlement entrance, middle part with burnt wall debris and third part with much less burnt wall debris at the backside (Neb-1) |
| 8a | II or III | two-part | on the narrow side short open empty part towards the settlement entrance followed by a long part |
| 8b | II or III | two-part | Like 8a but longer low magnetised part towards the entrance |
| 9 | II | two-part | exterior walls are displayed as highly magnetised stripes, internal division into two equally sized parts, on highly magnetised and one lower magnetised part |
