# Supplementary material for: Governing Tripolye: Integrative architecture in Tripolye settlements
Source: PLoS One. 2019 Sep 25;14(9):e0222243. doi: 10.1371/journal.pone.0222243 (PMC6760824; doi:10.1371/journal.pone.0222243)
Supplement: S1 File — (DOCX) [file pone.0222243.s003.docx]

# **S1 file. Maidanetske, trench 111, OxCal codes and results of Bayesian modelling**

## **OxCal Codes**

Plot("mega-structure 3")

{

Sequence("Maidanetske mega-structure 3")

{

Boundary( "start pits phase 1 pre-mega-structure");

Phase("pits phase 1 pre-mega-structure")

{

R_Date("Poz-87601",5020,35);

R_Date("Poz-87602",4955,30);

Interval("duration pits phase 1 pre mega-structure");

};

Boundary( "start levelling pre-mega-structure");

Phase("levelling pre-mega-structure")

{

R_Date("Poz-87599",5010,35);

R_Date("Poz-87600",4970,30);

Interval("duration levelling pre mega-structure");

};

Boundary( "start pits phase 2 pre-mega-structure");

Phase("pits phase 2 pre-mega-structure")

{

R_Date("Poz-87603",4990,35);

R_Date("Poz-87604",5000,35);

//R_Date("Poz-87605",5035,35);

Interval("duration pits phase 2 pre mega-structure");

};

Boundary("pre-mega-structure-mega-structure");

Phase("mega-structure")

{

//R_Date("Poz-87610",5035,35);

R_Date("Poz-87598",4990,35);

Interval("duration mega-structure");

};

Boundary("end mega-structure");

Phase("post-mega-structure")

{

R_Date("Poz-87721",4900,40);

// R_Date("Poz-87609",5055,35);

Interval("duration post mega-structure");

};

Boundary("end post mega-structure");

};

};

## **Results of Bayesian modelling**

A_model_=112.6
A_overall_=119.9

| Phase | Event | 68.2% | 95.4% | median | highest |
| --- | --- | --- | --- | --- | --- |
| pre-mega-structure | start pit phase 1 | 3794–3739 | 3812–3716 | 3770 | 3770 |
|  | duration pit phase 1 | 0-18 | 0–56 | 8 | 5 |
| mega-structure  construction and use | start levelling phase | 3780–3732 | 3788–3716 | 3756 | 3765 |
|  | duration levelling phase | 0–15 | 0–37 | 7 |  |
|  | start pit phase 2 | 3767 (9.2%) 3761  3748 (59.0%)3716 | 3781–3711 | 3742 | 3735 or 3720 |
|  | duration pit phase 2 | 0–15 | 0–40 | 7 | 20 or 5 |
|  | start use mega-structure | 3715–3707 | 3776–3703 | 3729 | 3715 |
|  | duration use-mega-structure | 0–21 | 0–55 | 10 | 5 |
| post-mega-structure | start post mega-structure activities | 3738–3690 | 3773–3673 | 3713 | 3710 |
|  | duration post-mega-structure activities | 0 –25 | 0 –78 | 11 | 15 |
|  | end post mega-structure activities | 3739 (4.6%)3731  3726 (63.6%)3661 | 3773 –3629 | 3698 | 3695 |

## **Interpretation**

Pre-mega-structure: 3770-3756 BCE

Mega-Structure: 3756-3713 BCE

Post-mega-structure 33713-3698 BCE
